# Periodontitis and MASLD: a narrative review of the direct oral-hepatic pathway

**DOI:** 10.3389/fcimb.2026.1865486

**Published:** 2026-07-08

**Authors:** Hai He, Zishuai Chen, Hengxiang Liu, Xiaohua Zhang, Zhiqiang Li, Tianzhu Song, Zifan Wang

**Affiliations:** 1 Key Laboratory of Biotechnology and Bioengineering of State Ethnic Affairs Commission, Biomedical Research Center, School of Bioengineering Northwest Minzu University, Lanzhou, Gansu, China; 2 School of Stomatology, Key Laboratory of Oral Diseases of Gansu Province, Key Laboratory of Stomatology of State Ethnic Afairs Commission, Northwest Minzu University, Lanzhou, Gansu, China

**Keywords:** direct oral hepatic pathway, hepatic steatosis, liver fibrosis, MASLD, periodontitis

## Abstract

Metabolic dysfunction-associated steatotic liver disease (MASLD) affects over 30% of adults globally, yet no pharmacotherapies are approved for its early stages. Emerging evidence from preclinical models and limited observational studies suggests that periodontitis may represent a biologically plausible, modifiable risk factor for MASLD through proposed direct oral--hepatic pathways; however, causal relationships remain to be established, and these pathways are currently insufficiently validated in humans. This narrative review synthesizes evidence ranging predominantly from *in vitro* and animal studies to limited human observational and interventional data consistent with three proposed mechanistic pathways by which periodontal pathogens and their derivatives may potentially contribute to MASLD pathogenesis: (i) disruption of hepatocyte lipid homeostasis through TLR4-mediated inflammatory signaling, (ii) amplification of hepatic inflammation via activation of Kupffer cells and liver sinusoidal endothelial cells, and (iii) promotion of fibrogenesis through hepatic stellate cell activation. We emphasize that these pathways remain largely hypothetical, derived principally from preclinical models, and await robust validation in human populations. Preclinical studies further suggest that reactive oxygen species (ROS) and associated inflammasome signaling—particularly NLRP3 pathway activation—may function as interconnected integrative nodes linking these processes, although the relative contribution of NLRP3-mediated mechanisms within this proposed framework remains to be systematically defined. Clinical evidence is currently limited to a single small prospective interventional study (n = 40) reporting that periodontal treatment may improve hepatic steatosis and inflammatory markers in patients with comorbid periodontitis and MASLD; these preliminary findings should be regarded as hypothesis-generating rather than confirmatory, and require independent replication in larger, adequately powered randomized controlled trials before any definitive clinical conclusions can be drawn. We critically appraise the current evidence base, highlighting the predominance of monomicrobial Porphyromonas gingivalis models, the lack of standardized methodologies, and the absence of human interventional studies capable of distinguishing direct from gut-mediated pathways. We explicitly note that the proposed oral-hepatic mechanisms are predominantly supported by preclinical data and remain insufficiently validated in humans, and we propose a standardized framework to advance causal inference and clinical translation.

## 1 Introduction

Metabolic Dysfunction-Associated Steatotic Liver Disease (MASLD), formerly known as Non-Alcoholic Fatty Liver Disease (NAFLD), is the updated definition of chronic liver disease set out in the clinical practice guidelines jointly published in 2023 by the European Association for the Study of the Liver (EASL) and the American Association for the Study of Liver Diseases (AASLD) and the Latin American Association for the Study of the Liver (ALEH). Its core diagnostic criteria comprise hepatic steatosis combined with metabolic dysfunction, and it is currently the chronic liver disease with the highest prevalence worldwide (Rinella et al., 2023; Rinella and Sookoian, 2024). As of 2021, the global prevalence of MASLD among adults had reached 32.4%, whilst the prevalence of the condition among Asian populations ranged between 28% and 32% (Yang et al., 2024; Ginès et al., 2025). The progression of MASLD is characterised by its gradual nature, advancing from simple hepatic steatosis to Metabolic dysfunction-Associated Steatohepatitis (MASH), liver fibrosis and cirrhosis, ultimately leading to end-stage liver disease and hepatocellular carcinoma (HCC); simultaneously, it significantly increases the risk of extrahepatic complications such as type 2 diabetes (T2DM) and cardiovascular disease, resulting in a continuously mounting global disease burden and public health pressure (Kendall et al., 2023; Lekakis and Papatheodoridis, 2024; James et al., 2025).

There remains a significant unmet clinical need in the management of MASLD: although the Food and Drug Administration (FDA) and the European Medicines Agency (EMA) have approved resmetirom and semaglutide for the treatment of non-cirrhotic MASLD with liver fibrosis (stages F2–F3) in 2024–2025, there are currently no approved curative treatments for early-stage MASLD (simple steatosis) (Malandris et al., 2026; Rinella and Sookoian, 2026). Current first-line clinical interventions continue to focus primarily on lifestyle interventions such as dietary control and exercise; however, long-term patient adherence remains consistently below 30%, making it impossible to effectively halt disease progression in the majority of the population (Caro-Sabido and Larrosa-Haro, 2019; Chen et al., 2025). The pathogenesis of MASLD is complex and multifactorial. In addition to classic aetiological factors such as metabolic disorders, insulin resistance and genetic susceptibility, growing evidence in recent years suggests that chronic infections originating in extrahepatic organs and persistent low-grade inflammation are important, modifiable risk factors in the onset and progression of MASLD (Schnabl et al., 2025; Solleiro-Villavicencio et al., 2025). Consequently, the identification of novel pathogenic targets and modifiable pathways in MASLD has become a central research focus and a pressing clinical need in the field of hepatology.

Periodontitis is one of the most common chronic infectious diseases worldwide. Data from the Global Burden of Disease (GBD) study indicates that over 1 billion people worldwide are affected by the condition, placing it among the most prevalent diseases globally. Specifically, the prevalence of severe periodontitis among adults worldwide stands at 11.2%, with the number of new cases rising from approximately 50.82 million to 89.61 million between 1990 and the year 2021, representing an increase of 76.32% (Hashim et al., 2025; Wang et al., 2025). The core pathological features of periodontitis are local chronic inflammation caused by the colonisation of periodontal pathogens, the destruction of the periodontal epithelial barrier, and the formation of periodontal pockets, which can lead to irreversible resorption of the periodontal connective tissue and alveolar bone; simultaneously, the compromised periodontal barrier allows periodontal pathogens and their by-products to continuously enter the bloodstream, triggering systemic low-grade inflammation and recurrent low-load bacteraemia; this also constitutes the core pathological basis for the impact of periodontitis on multiple organ systems throughout the body (Hajishengallis, 2015; Herrera et al., 2024). The biological mechanisms linking periodontal disease to systemic conditions including low-grade bacteremia, endotoxemia, and chronic inflammatory mediator release have been extensively characterized in the context of diabetes mellitus, cardiovascular disease, and chronic kidney disease (Sanz et al., 2018a; Sanz et al., 2018b; Kim and Pang, 2025). These shared pathways underscore that periodontal inflammation represents a common modifier of systemic disease risk. However, the specific application and validation of these mechanisms in MASLD pathogenesis remains comparatively underexplored.

In recent years, both observational studies and Mendelian randomisation analyses have suggested a close association between periodontitis and MASLD, supporting the possibility of a causal link between the two (Tan et al., 2024; Juzbašić et al., 2025). A cross-sectional study has shown that the prevalence of periodontitis is significantly higher in patients with MASLD than in the general population, and that the severity of periodontitis is significantly positively correlated with the grade of hepatic steatosis, the level of inflammation, and the stage of fibrosis in MASLD (Roesch-Ramos et al., 2026). Bidirectional cohort studies further suggest that baseline periodontitis is associated with future MASLD development and disease progression, although residual confounding cannot be fully excluded. Patients with severe periodontitis show significantly elevated risks of MASH and progressive liver fibrosis compared to periodontally healthy individuals, with associated risk multiples typically ranging from 1.6- to 3.7-fold in clinical studies. Notably, the association with liver fibrosis demonstrates a strength of approximately 2.3-fold (Hatasa et al., 2021; Kuroe et al., 2021; Åberg and Helenius-Hietala, 2022). With regard to this potential causal link, the vast majority of existing studies and reviews adopt the ‘gut-liver’ axis as their central explanatory framework. They propose that periodontal pathogens enter the gut via the oral cavity, compromise the integrity of the intestinal barrier, and trigger dysbiosis and enteric endotoxemia, which subsequently modulate pathological processes in the liver via the portal venous system (Tripathi et al., 2018).

Although existing research has made some progress, three key shortcomings remain in this field, severely limiting our comprehensive understanding of the association between periodontitis and MASLD and its clinical translation: firstly, over 90% of existing mechanistic studies rely excessively on indirect pathways mediated by the gut-liver axis, which cannot fully explain the independent causal relationship between the two; In recent years, numerous animal experiments and clinical studies have shown that the effect of periodontitis in exacerbating MASLD may occur, at least in part, independently of alterations in gut microbiota composition and intestinal barrier function, consistent with the hypothesis of a direct oral–hepatic pathway that does not rely on intestinal mediation (Nagasaki et al., 2020); however, this pathway has long been overlooked within the field. Secondly, evidence that periodontal pathogens and their metabolites directly regulate hepatic pathological processes is highly fragmented. Existing studies have only sporadically reported *in vitro* effects of individual periodontal bacterial strains on specific liver cell types, lacking systematic integration and critical analysis of evidence across the entire chain of direct pathways (Nagasaki et al., 2020; Dioguardi et al., 2025). Thirdly, existing research is unable to quantify the relative contribution of direct pathways to the association between periodontitis and MASLD. There is a lack of gold-standard research models capable of fully distinguishing between direct and indirect effects, and there are no unified experimental design protocols or detection standards. Consequently, the results of different studies exhibit poor reproducibility, preventing the formation of a consensus within the field (Dioguardi et al., 2025; Liu et al., 2025).

Accordingly, this review focuses on proposed mechanistic pathways by which periodontal pathogens and their derivatives may contribute to the onset and progression of MASLD. Systematic examination is provided of the biological basis, molecular regulatory networks and clinical translational evidence underpinning this pathway. We critically analyse the core limitations and methodological shortcomings of existing research, establish a standardised framework for future research in this field, and propose actionable directions, thereby offering a conceptual framework for hypothesis generation and future research for the prevention, early screening and targeted intervention of MASLD.

### 1.1 Literature Sources and Selection

A systematic search of PubMed (MEDLINE), Web of Science, Embase, and Cochrane Library was conducted from inception to December 2024. Search strings combined MeSH terms and free-text keywords across three domains: periodontal disease (periodontitis, Porphyromonas gingivalis, gingipains, outer membrane vesicles), MASLD/NAFLD (metabolic dysfunction-associated steatotic liver disease, non-alcoholic fatty liver disease, MASH, hepatic steatosis, liver fibrosis), and direct oral–hepatic mechanisms (hematogenous dissemination, bacteremia, lipopolysaccharide, endotoxemia).

Inclusion criteria were: (i) peer-reviewed studies reporting direct (non-gut-mediated) effects of periodontal pathogens or their derivatives on hepatocytes, Kupffer cells, hepatic stellate cells, or liver sinusoidal endothelial cells; (ii) *in vitro*, *in vivo* (with gut-interference controls), or human clinical/histopathological evidence; (iii) English-language publications. Exclusion criteria were: (i) studies exclusively addressing gut-liver axis mechanisms without direct pathway evidence; (ii) non-peer-reviewed sources; (iii) case reports lacking mechanistic insight.

Retrieved records were screened through duplicate removal, title/abstract review, and full-text assessment, with priority given to mechanistic studies employing gut-interference controls.

## 2 The biological basis and evidence-based data for the direct migration of periodontal pathogens to the liver

Before examining the biological mechanisms, it is critical to delineate the anatomical boundary between the direct migration pathway and the classical gut-liver axis pathway. The classical gut-liver axis posits that periodontal pathogens enter the gastrointestinal tract via the oral cavity, colonize the intestine, disrupt intestinal barrier integrity, and subsequently translocate to the liver via the portal venous system (Lei et al., 2023). In contrast, this chapter examines the putative direct haematogenous pathway: periodontal pathogens translocate from the periodontal pocket across the compromised epithelial barrier into the bloodstream (bacteraemia), potentially reach the liver via the systemic circulation (hepatic artery), and may directly colonize liver tissue without obligatory intestinal transit or colonization (Liu et al., 2023). It is important to note that tail-vein and jugular-vein injection models introduce bacteria directly into the systemic venous circulation, thereby routing them necessarily to the liver via the hepatic artery by experimental design; however, gingival inoculation models do not inherently discriminate between arterial and portal venous delivery unless coupled with gut-interference controls (e.g., portal-vein catheterization, enteric diversion, or germ-free animals). All evidence presented herein is selected specifically to support this non-enterogenic direct pathway, while acknowledging that the relative contribution of the hepatic artery versus portal venous recirculation under physiological conditions remains incompletely resolved. **Table 1** provides a hierarchical evaluation of the current evidence supporting direct hepatic colonization by periodontal pathogens, categorizing studies by evidence level, experimental model, and methodological approach.

**Table 1:** Hierarchical evidence for direct hepatic colonization of periodontal pathogens and route characteristics.

Study design	Study type	Representative study	Key findings	Sample/model	Detection method	Generalizability	Limitations	Reference
Cat-A	Tail-vein injection model	Sasaki et al., 2018	*Pg*-LPS directly induces hepatic steatosis and glucose/lipid metabolic disorders	C57BL/6 mice	PCR, histochemistry, biochemical assays	Low (animal)	Bypasses oral barrier selective pressure; high-dose endotoxin	(Sasaki et al., 2018)
Cat-A	Dental pulp infection model	Ishikawa et al., 2013	Oral *Pg* disseminates to liver via bloodstream, regulates hepatic glycogen synthesis through Akt/GSK-3β pathway	Mice	Fluorescent labeling, immunohistochemistry, Western blot	Low (animal)	Species differences; mouse model	(Ishikawa et al., 2013)
Cat-A	Gingival inoculation + *in vivo* imaging	Cheng et al., 2025	Cy7-labeled *Pg* migrates from gingiva to liver, enriches in hepatic tissue with prolonged retention	Mice	*In vivo* imaging, PCR, immunofluorescence	Low (animal)	Bacterial viability status to be confirmed	(Cheng et al., 2025)
Cat-A	Jugular vein injection model	Sasaki et al., 2018	Live *Pg* (OMZ314, Type II fimA) via jugular vein directly causes hepatic steatosis	C57BL/6J mice	Histology, biochemical assays	Low (animal)	Non-physiological infection route	(Sasaki et al., 2018)
Cat-H	Human liver biopsy sequencing	Yoneda et al., 2012	*Pg* detected in liver tissue of NASH patients (fimA genotyping), invasive genotypes account for 94.3%	Liver biopsy samples (MASH vs. controls)	fimA genotyping, sequencing	Moderate (human)	Cannot completely exclude gut origin	(Yoneda et al., 2012)
Cat-H	Serum antibody-metabolism association	Hatasa et al., 2021	Serum *Aa* antibody titers significantly correlate with hepatic steatosis severity and metabolic abnormalities in MASLD patients	MASLD patient cohort	ELISA, liver ultrasonography	Moderate (human)	Cross-sectional design; cannot establish causal temporality	(Hatasa et al., 2021)
Cat-H	Oral-liver microbiome association	Yang et al., 2023	Liver microbiota in HCC patients highly concordant with tongue dorsum microbiota	HCC patients (liver tissue + oral samples)	16S rRNA sequencing, FISH	Moderate (human)	Tumor tissue-specific; not MASLD	(Yang et al., 2023)
Cat-C	Hepatic abscess case report	Soeiro et al., 2019	*Dialister pneumosintes* (oral commensal) isolated from hepatic abscess	Clinical case (hepatic abscess)	Bacterial culture, 16S rRNA identification	Very low (single case)	Rare infection; not typical MASLD presentation	(Soeiro et al., 2019)
Cat-C	Hepatic abscess case report	Livingston & Perez-Colon, 2014	*Streptococcus intermedius* bacteremia following dental cleaning leads to hepatic abscess	Clinical case	Blood culture, imaging	Very low (single case)	Case report; infection rather than metabolic disease	(Livingston and Perez-Colon, 2014)

Evidence is categorized by study design type rather than hierarchical strength. Cat-A (animal models) includes experimental systems that exclude intestinal confounders, offering strong experimental control but limited applicability to human disease. Cat-H (human observational studies) includes clinical and epidemiological data with greater relevance to human disease, but these designs cannot fully exclude gut-mediated translocation or confounding variables. Cat-C (case reports) provides indirect clinical evidence. Animal models and human studies offer complementary strengths: the former provides mechanistic insight under controlled conditions, while the latter provides clinical relevance; neither type alone establishes causality in humans. Standard OCEBM levels: Level 1, systematic reviews and RCTs; Level 2, cohort studies; Level 3, case-control studies; Level 4, case series; Level 5, expert opinion and animal studies.

### 2.1 From periodontium to liver: barrier breakdown, haematogenous dissemination, and hepatic colonization

#### 2.1.1 Barrier compromise and bacterial entry: the initiation of bacteraemia

The migration of periodontal pathogens across the gingival epithelium is primarily driven by active enzymatic degradation of junctional proteins (Katz et al., 2002). *Porphyromonas gingivalis* (*Pg*), one of the most well-studied periodontal pathogens, secretes gingipains (HRgpA, RgpB, and Kgp) that directly proteolyze epithelial junctional proteins—including E-cadherin of adherens junctions and occludin of tight junctions—in gingival epithelial cells (Katz et al., 2002). This active proteolytic activity disassembles intercellular junctional complexes, facilitating bacterial translocation into the subepithelial vasculature independent of mechanical disruption (Katz et al., 2002). In addition, passive mechanical disruption from mastication, toothbrushing, or dental procedures can transiently breach the epithelial barrier, contributing to intermittent bacteraemia (Martins et al., 2024). Critically, these manipulations induce bacteraemia in a periodontal inflammation-dependent manner: the magnitude and duration of bacteraemia are significantly higher in individuals with periodontitis versus healthy periodontium, establishing that periodontal disease is the essential pathological prerequisite for sustained bacterial dissemination (Dhotre et al., 2018).

#### 2.1.2 Anatomical pathway: systemic circulation to hepatic sinusoids

**Figure 1:**
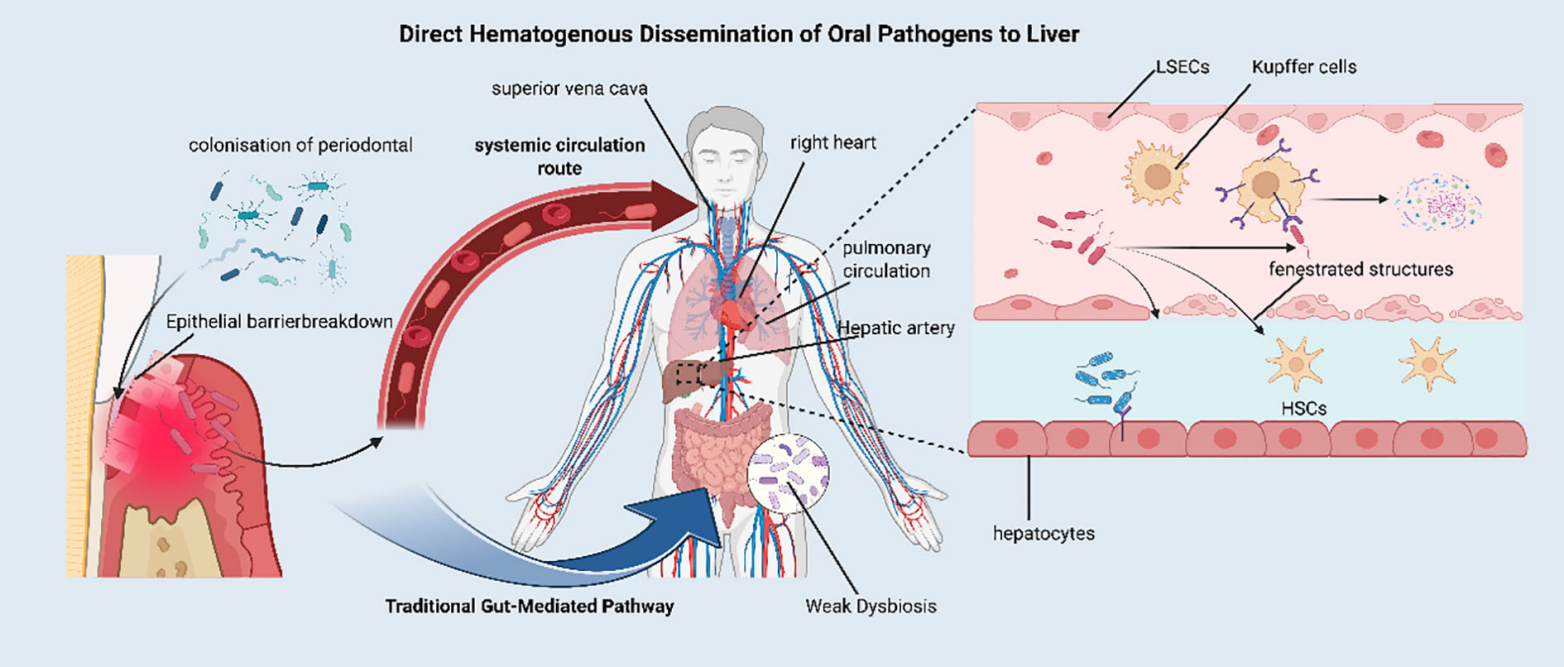
Direct hematogenous dissemination of periodontal pathogens to the liver independent of the gut-liver axis. Following gingipain-mediated disruption of the gingival epithelial barrier, periodontal pathogens may enter the bloodstream and reach the liver via the hepatic artery, potentially bypassing the traditional gut-mediated route. While direct hematogenous dissemination is biologically plausible and supported by experimental models, the relative contribution of the hepatic artery versus portal venous recirculation under physiological conditions—and the extent to which bacteria may simultaneously transit the intestine—remains to be fully quantified through gut-interference and portal-vein sampling studies. Leveraging the fenestrated architecture of liver sinusoidal endothelial cells, bacteria and their virulence derivatives translocate into the space of Disse to directly activate Kupffer cells, disrupt hepatocyte lipid homeostasis, and stimulate hepatic stellate cell (HSCs) activation, thereby illustrating the proposed direct oral–hepatic pathway for periodontitis-associated MASLD pathogenesis.

Once periodontal pathogens enter the bloodstream through the compromised periodontal barrier, they follow a specific anatomical pathway to reach the liver (Lei et al., 2023). First, these pathogens enter the superior vena cava through the facial vein and internal jugular vein, then pass through the right heart to complete the pulmonary circulation, exit the left heart to enter the systemic circulation, and ultimately colonize the hepatic tissue via the branches of the hepatic artery (**Figure 1**). In intravascular injection models (e.g., tail-vein or jugular-vein administration), this hematogenous transmission pathway necessarily bypasses the portal venous system and the gastrointestinal tract by experimental design (Kirvalidze and Gandhi, 2020; Dioguardi et al., 2025). Under physiological conditions, however, gingival inoculation models cannot definitively exclude that a fraction of bacteria may be swallowed, transit the intestine, and reach the liver via the portal vein; therefore, complete discrimination between the hepatic artery and portal venous routes requires additional gut-interference controls that are currently lacking.

#### 2.1.3 Hepatic tropism: structural and functional basis for bacterial capture

The liver possesses unique structural features that facilitate the capture and translocation of circulating microorganisms (Nature Immunology, 2013). The fenestrated structure and lack of a continuous basement membrane of liver sinusoidal endothelial cells (LSECs) create favorable conditions for the transport of bacterial products (such as lipopolysaccharide (LPS) and outer membrane vesicles (OMVs)). Leveraging this structural advantage, these bacterial products can enter the space of Disse through endocytosis or intercellular pathways, thereby making direct contact with hepatocytes and HSCs. Under specific pathological conditions, the barrier function of LSECs is impaired, and their transport efficiency of bacterial products is significantly enhanced, which in turn exacerbates local hepatic inflammatory responses and fibrotic processes (Boye et al., 2016; De Smedt et al., 2021) (**Figure 1**). Additionally, the liver represents the primary reticuloendothelial organ of the systemic circulation, with Kupffer cells accounting for over 80% of the body’s tissue-resident macrophages. These specialized macrophages are highly efficient at capturing circulating pathogens through pattern recognition receptors, further driving the hepatic tropism of haematogenous periodontal pathogens (Guilliams and Scott, 2022; Schuermans et al., 2024; Dioguardi et al., 2025). *In vivo* mouse experiments have confirmed that periodontal pathogens can colonize liver tissues via the systemic circulation, exhibiting the distinct characteristics of higher enrichment and longer retention in the liver. This distribution pattern supports the hypothesis that periodontal pathogens may exhibit tropism for liver tissues in experimental conditions (Cheng et al., 2025).

### 2.2 Evidence of hepatic colonisation in MASLD: complementary methodological evaluation

Current evidence supporting the direct hepatic migration of periodontal pathogens is categorized into two hierarchical levels ranked by complementary methodological strengths: (1) experimental evidence from animal models that exclude intestinal interference (Wang et al., 2022; Jin et al., 2026), and (2) correlative evidence from human studies utilizing strain-level genomic analysis to infer oral origin (Wang et al., 2022; Jin et al., 2026).

#### 2.2.1 Experimental evidence from animal models that exclude intestinal interference

Animal studies utilizing models that eliminate or bypass gut microbiota provide strong experimental support for direct haematogenous dissemination (Rush et al., 1989). In mouse models, intra-gingival injection or dental pulp infection with fluorescently labelled (Cy7) or genetically barcoded (SNAP-tagged) *Pg* resulted in the detection of viable bacteria, their DNA, and specific tracer signals in liver tissue via PCR, *in vivo* imaging, and immunohistochemistry. Concurrently, these models exhibited hepatic steatosis, impaired glycogen synthesis, fibrosis, and elevated inflammatory cytokine levels. Importantly, comparative oral gavage experiments demonstrated that *Pg* detection in the liver originated from direct hematogenous spread following oral cavity colonization, rather than through gastrointestinal translocation, providing supportive evidence for the oral origin of hepatic pathogens independent of gut microbiota mediation (Ishikawa et al., 2013; Leinwand et al., 2022; Cheng et al., 2025). However, these experiments compared gingival inoculation with oral gavage rather than systematically excluding intestinal transit; they therefore do not fully discriminate whether bacteria reached the liver exclusively via the hepatic artery following bacteraemia, or whether a subset was swallowed and subsequently recirculated through the portal venous system. Definitive route discrimination would require paired sampling of portal and systemic blood, or gut-interference controls (e.g., enteric diversion or portal-vein ligation), which remain to be systematically implemented.

#### 2.2.2 Correlative evidence from human studies

Clinical studies based on liver biopsy samples have provided correlational evidence that signals related to *Pg* can be detected in the liver tissues of patients with MASLD, and positive patients exhibit significantly higher liver fibrosis progression and scores compared to healthy populations. However, these observational findings cannot establish whether detected bacterial signals represent viable colonization, transient DNA deposition, or translocation artifacts, nor can they distinguish direct hematogenous dissemination from gut-mediated portal venous delivery. Serum antibody titers against *Aggregatibacter actinomycetemcomitans* (Aa) are closely associated with metabolic abnormalities and the degree of hepatic steatosis in MASLD patients, suggesting its potential involvement in disease pathogenesis. Notably, elevated antibody titers reflect systemic immune exposure rather than confirming direct hepatic infection; alternative explanations include shared inflammatory milieu or cross-reactive epitopes. Additionally, *Fusobacterium nucleatum* (Fn) has also been reported to be associated with MASLD progression in observational studies; the enrichment of Fn in oral samples from patients with advanced MASLD (such as those complicated with hepatocellular carcinoma) is significantly higher than that in early-stage patients and healthy controls, and its serum antibody levels are also correlated with abnormal lipid metabolism in these patients (Furusho et al., 2013; Hatasa et al., 2021; Dioguardi et al., 2025; Yang et al., 2025). These associations remain correlative; no interventional or mechanistic human studies have demonstrated that *Fn* directly modulates hepatic metabolic pathways independent of gut-mediated effects. Further 16S rRNA gene sequencing and fluorescence *in situ* hybridization (FISH) assays revealed that specific microorganisms (such as *Streptococcus* and *Stenotrophomonas*) detected in liver tissues of certain hepatocellular carcinoma patients exhibited high concordance with bacterial signatures in their oral (tongue surface) samples (Yang et al., 2023). While these findings are consistent with the hypothesis that oral microbiota may translocate to hepatic tissues, they cannot distinguish viable colonization from passive DNA transfer, nor can they exclude gut-mediated portal venous delivery as the primary route of hepatic bacterial detection. The interpretation that tongue dorsum microbiota serve as an important source of liver tissue microbiota therefore remains speculative. In the absence of alternative infectious sources, the clear clinical temporal association between typical oral commensal bacteria (such as *Dialister pneumosintes* and *Streptococcus intermedius*) isolated from liver abscesses and prior or concurrent periodontal infections in patients is consistent with the hypothesis that these pathogens may have migrated to the hepatic tissue via hematogenous dissemination from the oral primary focus (Livingston and Perez-Colon, 2014; Soeiro et al., 2019). However, these isolated case reports represent anecdotal evidence in acute infectious contexts rather than proof of chronic colonization in MASLD pathogenesis; they cannot exclude transient intestinal transit as an alternative route, nor do they establish that similar dissemination occurs in the absence of frank abscess formation. Additionally, multiple studies have detected DNA of *Pg* in the liver tissues of patients with MASLD, with bacterial loads demonstrating a positive correlation with the severity of hepatic fibrosis (Mei et al., 2024). While these findings are consistent with the hypothesis that this pathogen may systemically translocate to the liver via the bloodstream, potentially delivering virulence factors such as gingipains through OMVs, the presence of bacterial DNA alone does not demonstrate viable colonization, active virulence factor delivery, or causal contribution to fibrosis progression in human disease. Experimental models have shown that OMVs can reach hepatic tissue and activate immune responses (Lv et al., 2025), but whether this occurs at pathophysiologically relevant concentrations in human MASLD remains unverified.

In summary, convergent findings from experimental evidence support the biological plausibility of direct hematogenous dissemination of periodontal pathogens to the liver. Intravenous injection models—including tail-vein injection of sonicated *Pg* and jugular-vein injection of live *Pg* (OMZ314 strain, Type II fimA) in C57BL/6J mice (Sasaki et al., 2018)—introduce bacteria or their products directly into the systemic venous circulation, from which they necessarily reach the liver via the hepatic artery, thereby excluding the portal venous route by experimental design. Human oral sample fimA genotyping analysis (revealing that invasive genotypes accounted for 94.3% in MASH patients) (Yoneda et al., 2012) provides correlative evidence consistent with—but not demonstrative of—oral-hepatic bacterial transmission. Nevertheless, these models serve as proof-of-concept rather than route-fidelity evidence: intravenous injections prove that periodontal pathogens can reach the liver via the systemic circulation under artificial conditions, but do not replicate the oral barrier’s selective pressure or quantify the relative contribution of the hepatic artery versus portal vein under natural infection conditions. Gingival inoculation models more closely approximate physiological bacteraemia, yet without portal-vein versus systemic blood sampling or gut-interference controls, they cannot definitively distinguish whether hepatic bacteria arrived via the hepatic artery, portal venous recirculation, or both. Building upon the experimental evidence that periodontal pathogens may reach the liver through systemic circulation and induce pathological alterations in preclinical models, these findings generate a testable hypothesis for a direct causal relationship between periodontal infection and hepatic metabolic diseases, independent of gut microbiota mediation. However, this hypothesized mechanism remains to be validated in human interventional studies capable of distinguishing direct from gut-mediated effects (Yoneda et al., 2012; Sasaki et al., 2018).

## 3 Proposed mechanistic pathways by which periodontal pathogens and their derivatives may contribute to MASLD pathogenesis

Before examining specific cellular targets, it is essential to establish the operational definitions governing this chapter. In the context of this review, “direct” mechanisms encompass three hierarchical levels: (i) anatomical directness—pathogens reach the liver via the hepatic artery (systemic circulation) without obligatory intestinal colonization or portal venous transit, as established in Chapter **2**; (ii) cellular directness—physical interaction between bacterial components (live bacteria, LPS, OMVs, gingipains) and liver resident cells; and (iii) mechanistic directness—effects occurring within the liver parenchyma, distinguished from systemic indirect effects mediated by gut microbiota remodeling. Notably, intrahepatic paracrine signaling (secondary activation via cytokine relay between liver cells) constitutes a downstream event of the direct pathway, distinct from gut-mediated indirect effects.

As delineated in Chapter **2**, periodontal pathogens traversing the hepatic artery sequentially encounter: (i) the fenestrated LSECs and Kupffer cells lining the sinusoids, (ii) hepatocytes across the Disse space, and (iii) perisinusoidal HSCs. This anatomical hierarchy dictates the temporal sequence of pathogenic events: endothelial activation and innate immune recognition precede parenchymal metabolic injury, whereas sustained inflammatory milieu creates the permissive niche for fibrogenic signaling to HSCs. **Table 2** presents a comprehensive overview of the molecular mechanisms by which periodontal pathogens and their derivatives directly regulate hepatic cellular targets, organized by pathogen, effector molecule, target cell, signaling pathway, and pathological outcome.

**Table 2:** Overview of proposed molecular mechanisms by which periodontal pathogens may contribute to MASLD pathogenesis.

Pathogen	Effector molecule	Target cell	Core pathway	Key molecular events	Pathological effect	Vicious cycle node	Evidence source	Evidence strength	Reference
*Pg*	LPS	Hepatocytes	TLR4→NF-κB/JNK	Upregulation of ACC1; downregulation of SREBP-1c; compensatory upregulation of PPARα (insufficient)	Hepatic steatosis	Lipid accumulation → mitochondrial ROS → inflammatory activation	*In vitro* (HepG2 cells); Animal (tail-vein injection, C57BL/6 mice)	Moderate (consistent across cell line and rodent models; HepG2 metabolic programming differs from primary hepatocytes; supraphysiological LPS doses used)	(Sasaki et al., 2018; Ding et al., 2019; Chihara et al., 2023)
*Pg*	Gingipains	Hepatocytes	ROS→NF-κB/HDAC1→SREBP-1c	Proteolytic degradation of INSR α-subunit; mitochondrial damage; oxidative stress	Insulin resistance + steatosis	Oxidative stress integration point (ROS hub)	*In vitro* (HepG2 cells, primary hepatocytes); Animal (chronic oral infection, high-fat diet models)	Moderate (reproduced across multiple *in vitro* systems; *in vivo* evidence from chronic infection models; no human histopathological confirmation of gingipain-mediated INSR degradation in liver)	(Prasun et al., 2021; Wang et al., 2022; Liu et al., 2024; Zhang et al., 2025)
*Pg*	OMVs	Kupffer cells	TLR4/MyD88→NF-κB	M1 polarization; NLRP3 inflammasome activation; metabolic reprogramming to aerobic glycolysis	Hepatic inflammatory cascade	Inflammation → hepatocyte injury → HSC activation	*In vitro* (RAW264.7 macrophages, primary Kupffer cells); Animal (intraperitoneal injection, C57BL/6 mice)	Low–Moderate (intraperitoneal injection does not replicate chronic low-grade bacteremia; no human studies quantified OMV accumulation in hepatic tissue or Kupffer cell activation markers post-periodontal therapy)	(Bellanti et al., 2025; Lv et al., 2025; Ma et al., 2025; Zhou et al., 2025)
*Aa*	LPS	Kupffer cells	TLR2/TLR4-MyD88	NF-κB nuclear translocation; NLRP3 activation; IL-1β maturation and release	Pro-inflammatory cytokine storm	Inflammatory amplifier	*In vitro* (macrophage cell lines)	Low (no hepatic Kupffer cell-specific studies with Aa; no gut-interference controls in available studies; extrapolation from periodontal macrophage data to hepatic context is speculative)	(Park et al., 2014; Zhang et al., 2019; Yue et al., 2021; Zhou et al., 2023; Okano et al., 2024)
*Fn*	Whole bacterial components	LSECs	TLR4-NF-κB/MAPK	Upregulation of ICAM-1 (CD54), VCAM-1 (CD106), E-selectin	Immune cell adhesion and intrahepatic infiltration	Inflammatory cell recruitment → inflammation amplification	*In vitro* (LSEC cell lines)	Low (single *in vitro* study; no *in vivo* hepatic dissemination models with gut-interference controls; applicability to chronic low-grade endotoxemia of periodontitis unverified)	(Mukherjee et al., 2025; Wang et al., 2025)
*Pg*	Gingipains	HSCs	PAR2→TGF-β1 autocrine/Smad2/ERK	Upregulation of TGF-β receptor II; Smad2 phosphorylation; α-SMA and type I collagen secretion	HSCs activation → myofibroblast transformation	Fibrogenesis lock-in mechanism (HSC-inflammation mutual maintenance)	*In vitro* (immortalized LX-2 HSCs, primary rat HSCs); Animal (odontogenic infection, high-fat diet MASH model)	Moderate (replicated in multiple HSC lines and primary cells; *in vivo* odontogenic model provides causal evidence in rodents; no human interventional studies assessed HSC activation markers post-periodontal therapy)	(Nagasaki et al., 2020; Nagasaki et al., 2021)
*Pg*	OMVs	Macrophages → HSCs (paracrine)	Thbs1/TGF-β signaling axis	Macrophage Thbs1 upregulation; activation of latent TGF-β; indirect HSC activation	Fibrosis progression (indirect pathway)	Paracrine amplification loop	*In vitro* (macrophage-HSC co-culture)	Low (co-culture system does not recapitulate hepatic lobule architecture; no *in vivo* validation of Thbs1-mediated paracrine HSC activation in periodontal-liver models; species-specificity of OMV effects unexplored)	(Seki et al., 2025; Lv et al., 2026)

Evidence strength classification: High = replicated across multiple independent *in vivo* models with gut-interference controls and supported by human observational or interventional data; Moderate = consistent across *in vitro* and at least one *in vivo* model, but lacking human validation or limited by methodological constraints (e.g., artificial delivery routes, transformed cell lines); Low = primarily *in vitro* or single-model evidence, with substantial extrapolation required to human disease context; Very Low, hypothetical mechanisms inferred from indirect evidence or analogous disease models. Vicious cycle nodes correspond to the three positive feedback loops described in Section 3.4: steatosis-inflammation loop, inflammation-fibrogenesis loop, and Reactive oxygen species (ROS) integration network. The NLRP3 inflammasome functions as a central convergence node across these loops: it integrates microbial stimulation (LPS, OMVs), metabolic stress (lipotoxicity, mtROS, mtDNA), and oxidative damage (mtDNA) to drive IL-1β/IL-18-mediated inflammatory amplification and HSC activation (Inzaugarat et al., 2019). All mechanisms in this table derive from monomicrobial *P. gingivalis* challenge models; no entry meets “High” evidence strength for the direct oral-hepatic pathway in humans. Direct experimental evidence for *F. nucleatum*, *A. actinomycetemcomitans*, *T. forsythia*, or *T. denticola* regulating these pathways through the direct oral-hepatic pathway is absent. Notably, while A. actinomycetemcomitans has been shown to activate NLRP3 in macrophages (Okano et al., 2024), direct evidence for NLRP3 activation by *F. nucleatum*, *T. forsythia*, or *T. denticola* within the hepatic microenvironment remains lacking. The generalizability of these mechanisms to polymicrobial periodontitis remains hypothetical.

### 3.1 Proposed contribution of periodontal pathogen derivatives to hepatocyte lipid metabolism dysregulation and steatosis: experimental evidence and translational uncertainty

The liver is the central organ in the body’s lipid metabolism (Bechmann et al., 2012); simple steatosis, resulting from an imbalance between lipid synthesis and catabolism in hepatocytes, represents the initial key step in the development of MASLD (Koo, 2013). While Pg dominates current literature (Juzbašić et al., 2025), this predominance reflects the availability of well-characterized virulence factors (gingipains, LPS, OMVs) and genetically tractable strains rather than the ecological reality of human periodontitis, which is fundamentally a polymicrobial dysbiosis (Hajishengallis, 2015; Hashim et al., 2025; Wang et al., 2025). Periodontal biofilms exhibit complex ecological interactions, including metabolic cross-feeding, co-aggregation, and quorum-sensing-mediated signaling among diverse species (Hajishengallis, 2015). However, direct experimental evidence for non-Pg periodontal pathogens—or polymicrobial consortia—regulating hepatocyte lipid metabolism via the direct oral-hepatic pathway remains critically lacking. *F. nucleatum* has been shown to activate TLR4-associated inflammatory signaling and promote oxidative stress and Kupffer cell activation within the liver (Park et al., 2014), and co-culture experiments suggest that *F. nucleatum* may directly promote lipid accumulation in hepatic cells (Zhou et al., 2023). Nevertheless, these studies did not incorporate gut-interference controls to exclude intestinal transit, nor did they assess hepatic lipid metabolic endpoints *in vivo*. *A. actinomycetemcomitans*, *T. forsythia*, and *T. denticola* have not been experimentally demonstrated to directly regulate hepatocyte lipid metabolism through hematogenous dissemination. Serum antibody titers against A. actinomycetemcomitans correlate positively with visceral adiposity and insulin resistance in MAFLD patients (Hatasa et al., 2021), but this observational association cannot establish direct hepatic effects or distinguish direct from gut-mediated pathways. The mechanisms described in this section therefore derive predominantly from Pg-based cell culture and rodent models; their generalizability to the polymicrobial context of human periodontitis and their relevance to human MASLD pathogenesis remain hypothetical and await validation in studies employing multi-species infection models with appropriate gut-interference controls.

#### 3.1.1 direct infection of hepatocytes by live periodontal pathogens has been shown in experimental models to induce lipid metabolism disorders

**Figure 2:**
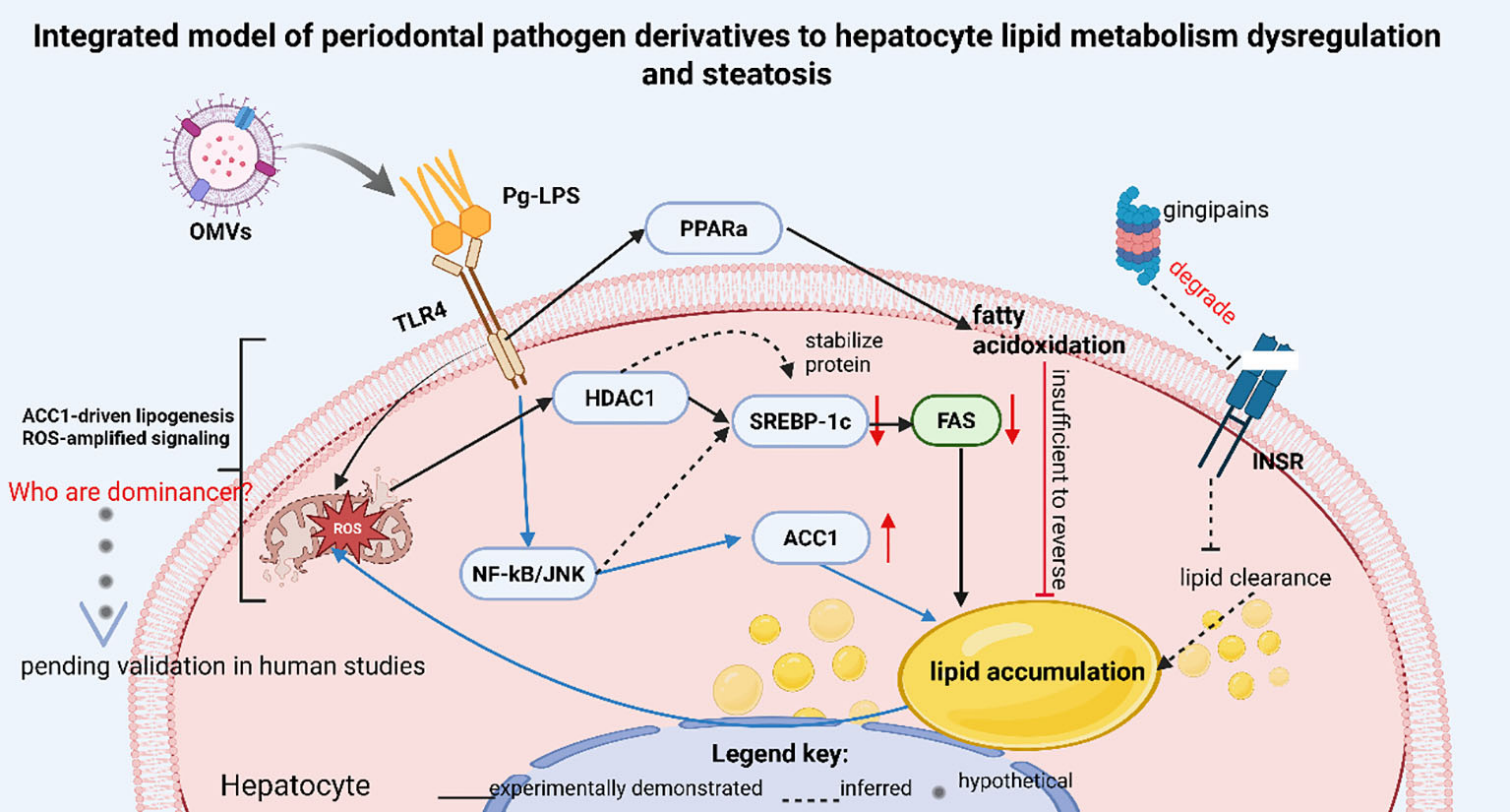
Integrated model of hepatocyte lipid metabolism dysregulation by periodontal pathogen derivatives. Solid lines indicate experimentally demonstrated pathways; dashed lines indicate inferred pathways; dotted lines indicate hypothetical pathways. The diagram illustrates how apparently contradictory findings are reconciled through a ROS-centric feed-forward loop. (1) [Experimentally demonstrated] Acute Pg-LPS/OMVs activate TLR4→NF-κB/JNK signaling, upregulating ACC1 independently of SREBP-1c in hepatocytes (Sasaki et al., 2018; Ding et al., 2019); (2) [Experimentally demonstrated] mitochondrial ROS generated under inflammatory stress activate NF-κB/HDAC1, which can stabilize SREBP-1c protein despite transcriptional downregulation (Guo et al., 2020); (3) [Inferred] the temporal switch from SREBP-1c suppression (chronic infection) to SREBP-1c activation (acute oxidative stress) represents an integrative interpretation across distinct experimental paradigms; (4) [Experimentally demonstrated] PPARα-mediated fatty acid oxidation is compensatorily upregulated but remains insufficient to reverse net lipid accumulation in preclinical models (Sasaki et al., 2018); (5) [Experimentally demonstrated *in vitro*] gingipains degrade the insulin receptor (INSR), inducing insulin resistance that further suppresses lipid clearance (Liu et al., 2024), although whether this occurs at pathophysiologically relevant concentrations in human liver tissue remains unverified. (6) [Hypothetical] The net pathological outcome-–hepatic steatosis-–reflects a proposed dominance of ACC1-driven lipogenesis and ROS-amplified lipogenic signaling over compensatory oxidative pathways, pending validation in human studies. Legend key: — experimentally demonstrated; - - - inferred; ··· hypothetical.

*In vitro* and animal-model studies have shown that *Pg* can act on hepatocytes via its virulence factors, modulating pathways related to lipid metabolism and disrupting lipid homeostasis under experimental conditions (Ding et al., 2019; Jia et al., 2023). Whether these effects occur in human liver tissue *in vivo* remains to be determined. Research on the human immortalised hepatocyte line HepG2 has provided a chain of experimental evidence consistent with this hypothesis. Upon co-incubation of HepG2 cells with *Porphyromonas gingivalis* lipopolysaccharide (*Pg*-LPS), the formation of lipid droplets within the hepatocytes increased significantly (Ding et al., 2019); this effect was even more pronounced in a microenvironment characterised by abnormal lipid metabolism (Ding et al., 2019). However, HepG2 cells represent a transformed cell line with altered metabolic programming compared to primary human hepatocytes, and the supraphysiological LPS concentrations typically employed in such experiments may not reflect circulating levels in human periodontitis. Animal experiments further demonstrated that administration of *Pg*-LPS under high-fat diet conditions has been shown to induce hepatic steatosis in rodent models, leading to the accumulation of lipid components such as triglycerides and free fatty acids in liver tissue (Chihara et al., 2023). Whether comparable LPS exposure occurs in human periodontitis, and whether the resulting hepatic effects mirror human MASLD pathophysiology, remains unknown. Mechanistically, the apparent contradictions in lipid metabolic findings across studies can be reconciled by considering differences in experimental models, exposure duration, and molecular regulatory nodes (**Figure 2**). These observations do not reflect a single unified pathway but rather distinct, temporally separated responses that converge on net hepatic lipid accumulation: (i) Model-dependent divergence: chronic infection versus acute LPS challenge. Chronic oral P. gingivalis infection in murine models downregulates the hepatic lipogenic transcription factor SREBP-1c and its downstream target fatty acid synthase (FAS) (Maekawa et al., 2011). This downregulation likely represents an adaptive host response to sustained bacterial colonization, wherein the liver suppresses *de novo* lipogenesis under chronic inflammatory stress. By contrast, direct Pg-LPS challenge via tail-vein injection—a model that bypasses oral barrier dynamics and introduces supraphysiological endotoxin loads—significantly upregulates acetyl-CoA carboxylase 1 (ACC1) and enhances fatty acid elongation metabolism (Sasaki et al., 2018). ACC1 catalyzes the rate-limiting step in fatty acid synthesis independently of SREBP-1c regulation; its upregulation under acute TLR4/NF-κB activation (Ding et al., 2019) may therefore promote lipogenesis even when SREBP-1c is suppressed. These divergent transcriptional responses (SREBP-1c↓ in chronic infection versus ACC1↑ in acute LPS exposure) are not mutually exclusive; they originate from different experimental paradigms that model distinct pathophysiological scenarios. (ii) Temporal dynamics: compensatory PPARα activation and its failure. Concurrent with ACC1 upregulation, acute Pg-LPS challenge elicits significant compensatory activation of the PPARα-mediated fatty acid oxidation pathway, characterized by marked upregulation of ACOTs, CPT1b, and the Cyp4a family (Sasaki et al., 2018). This represents a canonical hepatic stress response wherein PPARα-driven β-oxidation is recruited to counteract lipid overload. Nevertheless, this compensatory response remains quantitatively insufficient to reverse the progression of hepatic steatosis in these preclinical systems (Sasaki et al., 2018), suggesting that the rate of *Pg*-LPS-induced lipogenesis exceeds the capacity of induced fatty acid oxidation. Whether similar compensatory pathways are activated in human MASLD complicated by periodontitis, and whether they are similarly overwhelmed by chronic low-grade bacteremia, has not been investigated. (iii) Integrated interpretation. Collectively, these findings suggest a stage-dependent metabolic reprogramming: chronic periodontal infection may initially suppress SREBP-1c-mediated lipogenesis as an adaptive response, whereas acute bacteremic spikes (e.g., following dental procedures (Martins et al., 2024)) or direct LPS entry into the systemic circulation may override this adaptation through ACC1-dependent pathways. The net pathological outcome—hepatic steatosis—reflects the dominance of ACC1-driven lipogenesis and PPARα compensation failure over SREBP-1c suppression. The extent to which these temporally dynamic and model-specific transcriptional changes translate to human hepatocyte biology under physiological bacterial exposure remains to be determined. Moreover, all mechanistic evidence in this section derives from monomicrobial Pg challenge models. Polymicrobial periodontal infection models incorporating *Pg*, *T. forsythia*, *T. denticola*, and *F. nucleatum* have demonstrated intravascular dissemination to the heart and aorta (Chukkapalli et al., 2017; Gao et al., 2020; Ahn et al., 2021), but these studies did not assess hepatic lipid metabolism endpoints or include gut-interference controls. Consequently, whether polymicrobial dysbiosis exerts synergistic, additive, or antagonistic effects on hepatocyte lipid homeostasis through the direct oral-hepatic pathway remains entirely unknown.

#### 3.1.2 Experimental evidence for bacterial derivative-induced lipogenesis via mitochondrial redox dysregulation as a putative metabolic switch

*In vitro* and animal studies suggest that virulence factors and structural components secreted by periodontal pathogens may directly act on hepatocytes to induce lipid metabolic disorders in the absence of viable bacterial colonization (Lv et al., 2025). These findings establish biological plausibility but do not confirm that equivalent processes occur in human liver tissue at pathophysiologically relevant concentrations. The most extensively studied derivatives include *Pg*-LPS, gingipains, and bacterial OMVs (Lv et al., 2025).

Building upon the model-dependent metabolic framework outlined in Section 3.1.1, preclinical studies further suggest that reactive oxygen species (ROS) may function as a unifying molecular switch integrating the seemingly contradictory lipogenic and oxidative responses into a coherent pathogenic sequence (Zhang et al., 2025). Rather than treating ACC1 upregulation and PPARα activation as isolated events, emerging evidence positions ROS at the nexus of a feed-forward metabolic loop: Pg-LPS and gingipains induce mitochondrial damage and endoplasmic reticulum stress in hepatocytes, characterized by disorganized cristae and decreased mitochondrial density, leading to ROS accumulation (Wang et al., 2022; Ying et al., 2026). This mitochondrial dysfunction activates the redox-sensitive transcription factor NF-κB (Prasun et al., 2021; Zhang et al., 2025), which—through histone deacetylase 1 (HDAC1)-mediated stabilization—paradoxically enhances SREBP-1c activity (Guo et al., 2020), potentially overriding the chronic infection-induced SREBP-1c suppression described in Section 3.1.1 (Maekawa et al., 2011). Thus, ROS may serve as the mechanistic bridge between acute LPS-driven ACC1 upregulation and chronic inflammation-modulated SREBP-1c dynamics, establishing a self-amplifying cycle wherein lipid deposition generates ROS, and ROS drives further lipogenesis (Prasun et al., 2021; Zhang et al., 2025).

Critically, this ROS-centric integration helps explain why PPARα-mediated compensatory oxidation fails to prevent steatosis: ROS-induced NF-κB activation not only promotes lipogenesis but also impairs mitochondrial function (Prasun et al., 2021), thereby compromising the very oxidative machinery that PPARα seeks to upregulate (Sasaki et al., 2018). The compensatory PPARα response therefore operates within a redox-compromised cellular environment where its efficacy is intrinsically limited. No human studies have quantified whether periodontitis-derived ROS constitutes a quantitatively significant driver of hepatic redox dysregulation distinct from diet- or obesity-associated oxidative stress.

Preclinical studies suggest that ROS may function not only as a downstream consequence of metabolic disorders but also as an upstream modulator that promotes lipogenesis under experimental conditions by regulating key metabolic nodes such as AMPK, SREBP-1c, and PPARα, thereby potentially disrupting lipid homeostasis (Zhang et al., 2025). The extent to which ROS acts as a causative metabolic switch in human MASLD remains uncertain; moreover, the specific contribution of periodontitis-derived ROS to hepatic redox dysregulation, as distinct from diet- or obesity-associated oxidative stress, has not been quantified in human populations. *Pg*-LPS and gingipains have been shown in experimental systems to induce mitochondrial damage and endoplasmic reticulum stress in hepatocytes, characterized by disorganized cristae and decreased mitochondrial density, potentially leading to ROS accumulation and oxidative injury (Wang et al., 2022; Ying et al., 2026). This mitochondrial dysfunction activates the redox-sensitive transcription factor NF-κB (Prasun et al., 2021; Zhang et al., 2025), which further enhances SREBP-1c activity through histone deacetylase 1 (HDAC1)-mediated stabilization (Guo et al., 2020), potentially establishing a feed-forward loop wherein lipid deposition generates ROS, and ROS may drive further lipogenesis (Prasun et al., 2021; Zhang et al., 2025). Additionally, *in vitro* studies demonstrate that gingipains have been shown *in vitro* to degrade the α-subunit of the hepatocellular insulin receptor (INSR) through direct proteolytic activity, impairing insulin signal transduction and potentially inducing hepatocellular insulin resistance in experimental systems, thereby potentially promoting lipid synthesis and suppressing lipid degradation (Liu et al., 2024) (**Figure 2**). No human studies have confirmed that gingipains reach the liver at concentrations sufficient to proteolyze INSR *in vivo*, nor have clinical trials demonstrated that periodontal therapy improves hepatic insulin sensitivity through this specific mechanism.

Reconciling contradictory lipid metabolic findings: an integrated framework. In summary, the simultaneous description of SREBP-1c downregulation, ACC1 upregulation, PPARα activation, and net hepatic steatosis in the literature reflects four non-mutually exclusive dimensions of variation (**Table 2**): (i) experimental model—chronic oral infection (Maekawa et al., 2011) versus acute intravenous LPS challenge (Sasaki et al., 2018); (ii) disease stage—early adaptive SREBP-1c suppression versus late ROS-driven lipogenic rebound (Guo et al., 2020; Zhang et al., 2025); (iii) molecular specificity—ACC1 operates independently of SREBP-1c regulation, enabling parallel activation of distinct lipogenic nodes; and (iv) compensatory failure—PPARα-mediated oxidation is induced but overwhelmed by the rate of ROS-amplified lipogenesis (Sasaki et al., 2018). This framework does not resolve the contradictions but rather contextualizes them as complementary observations from different experimental paradigms, each modeling distinct aspects of the periodontitis-MASLD interface. All mechanisms described herein derive from monomicrobial *P. gingivalis* challenge models; whether polymicrobial periodontal infection produces qualitatively distinct metabolic reprogramming remains unknown.

Bacterial OMVs are nanoscale membrane-bound vesicles secreted by periodontal pathogens, capable of carrying various effector molecules including LPS, gingipains, and bacterial nucleic acids (Gui et al., 2016; Fan et al., 2023). These vesicles remain stable in the circulation and can directly reach the liver. Existing studies have demonstrated that OMVs derived from *Pg* can be transported to hepatic tissue without live bacterial infection, where they activate Kupffer cells and HSCs, inducing inflammatory responses and impairing insulin sensitivity in a dose-dependent manner (Wang et al., 2022; Lv et al., 2025). Furthermore, OMVs can evade host immune clearance in the circulation, and their pro-inflammatory effects persist significantly longer than those of free LPS (Svennerholm et al., 2017; Lv et al., 2025). It is important to note that the OMV-mediated mechanisms described herein are derived exclusively from *P. gingivalis*. Whether OMVs from *F. nucleatum*, *A. actinomycetemcomitans*, *T. forsythia*, or *T. denticola* exhibit comparable hepatic tropism, stability in circulation, and capacity to induce hepatocyte lipid metabolic dysregulation has not been investigated. Furthermore, it remains controversial whether periodontal pathogen-derived OMVs can be transferred to the liver at pathophysiologically relevant concentrations (Lv et al., 2025). The potential for inter-species OMV synergy within polymicrobial biofilms—wherein OMVs from different species may carry distinct virulence cargo and target different hepatic cell types—represents an entirely unexplored dimension of the direct oral-hepatic pathway.

### 3.2 Proposed direct activation of hepatic innate immunity and potential promotion of hepatic inflammatory cascades: preclinical evidence

Innate immune dysregulation in the liver represents the central driver in the progression of MASLD from simple steatosis to MASH (Meyer et al., 2024). The liver harbors the body’s most abundant population of innate immune cells, including Kupffer cells, LSECs, innate lymphoid cells (ILCs), dendritic cells (DCs), among others (Krenkel and Tacke, 2017; Kubes and Jenne, 2018). While the following sections emphasize *Pg*-derived mechanisms, it is critical to recognize that periodontal biofilms comprise polymicrobial communities with species-specific immunostimulatory profiles. *F. nucleatum* has been shown to activate TLR4-NF-κB/MAPK signaling in hepatic cells (Mukherjee et al., 2025), and *A. actinomycetemcomitans* activates TLR2/TLR4-MyD88 signaling in macrophages (Park et al., 2014). However, direct comparative studies examining whether non-Pg species activate Kupffer cells, LSECs, or other hepatic innate immune populations through the direct oral-hepatic pathway—with appropriate gut-interference controls—are absent from the literature. The ecological interactions within periodontal biofilms (e.g., metabolic cross-feeding between Pg and T. forsythia, co-aggregation of *F. nucleatum* with early colonizers) may modulate the immunostimulatory potential of individual species (Hajishengallis, 2015), yet whether such polymicrobial dynamics alter hepatic innate immune activation remains entirely speculative.

#### 3.2.1 Direct activation of hepatic Kupffer cells may induce pro-inflammatory responses

Kupffer cells, as liver-specific resident macrophages, serve as the first line of defense against blood-borne microbial products in the hepatic microenvironment (Dixon et al., 2013) and represent the core recognition and response cells for periodontal pathogen-derived outer membrane vesicles and lipopolysaccharides circulating in the bloodstream (Dioguardi et al., 2025; Lv et al., 2025). This proposed role is supported by *in vitro* and rodent model evidence (**Table 2**); human validation is currently unavailable. *In vitro* studies have shown that Aa can activate macrophages via the TLR2/TLR4-MyD88 signaling pathway (Park et al., 2014), whereas hepatic Kupffer cells in rodent models of periodontal infection exhibit activation of this pathway along with NF-κB nuclear translocation (Yue et al., 2021). Whether human Kupffer cells respond similarly to circulating periodontal pathogen products at physiological concentrations, and whether such activation contributes to MASH progression independent of gut-derived endotoxemia, remains speculative. Meanwhile, *Aa* has been shown to activate the NLRP3 inflammasome, promoting IL-1β release (Okano et al., 2024), and NLRP3 activation in Kupffer cells represents a critical mechanism underlying hepatic inflammation (Zhang et al., 2019). However, accumulating evidence positions the NLRP3 inflammasome not merely as a downstream consequence of TLR4 activation, but as an independent integrative node that transduces metabolic stress into inflammatory signals (Inzaugarat et al., 2019). In MASLD pathogenesis, multiple metabolic stimuli—including palmitate, cholesterol crystals, and endoplasmic reticulum stress—cooperatively prime NLRP3 expression via NF-κB signaling, while mitochondrial dysfunction provides the second signal necessary for inflammasome assembly (Ramos-Tovar and Muriel, 2023). Upon activation, NLRP3 drives caspase-1-mediated maturation of pro-IL-1β and pro-IL-18 into their biologically active forms (Knorr et al., 2023). IL-1β promotes hepatocellular lipid accumulation, recruits inflammatory infiltrates, and activates hepatic stellate cells, thereby driving the steatosis-to-inflammation-to-fibrosis cascade (Knorr et al., 2023). IL-18, distinct from IL-1β in its functional profile, participates in NK cell activation and IFN-γ production, contributing to MASH-associated inflammatory amplification (Knorr et al., 2023). Notably, myeloid cell-specific NLRP3 activation is particularly critical for the progression from MAFLD to fibrotic MASH (Knorr et al., 2023). Collectively, these findings suggest that periodontal pathogen products may regulate Kupffer cell function through NLRP3-dependent and NLRP3-independent axes, thereby mediating inflammatory responses in liver tissue.

*P. gingivalis*-mediated NLRP3 activation: TLR4-dependent priming and second signals. *P. gingivalis* LPS regulates the NLRP3 inflammasome through TLR4-dependent signaling, mediating inflammatory cytokine release and alveolar bone resorption in periodontitis (Hou et al., 2022). Synergistically, *Pg* LPS combined with IFN-γ significantly enhances NLRP3 pathway activation, while A20 gene silencing further exacerbates this process (Yang et al., 2021). Hypoxic conditions, mimicking the periodontal pocket microenvironment, combined with *Pg*-LPS, markedly enhance NLRP3 activation in human gingival fibroblasts (Yamaguchi et al., 2017). These findings establish that *Pg* products can prime and activate the NLRP3 inflammasome through TLR4-dependent mechanisms, with inflammatory and hypoxic microenvironments amplifying the response.

Redox-sensitive inflammatory signaling constitutes a critical amplifier of this process (Bellanti et al., 2025). Upon phagocytosing periodontal pathogen-derived OMVs (Zhang et al., 2024), Kupffer cells undergo metabolic reprogramming toward aerobic glycolysis (the Warburg effect) (Ma et al., 2025) with concomitant ROS generation (Bellanti et al., 2025; Lv et al., 2025) and pro-inflammatory M1 polarization (Bellanti et al., 2025; Ma et al., 2025). Mitochondrial ROS (mtROS) serves as an essential upstream signal for NLRP3 inflammasome assembly: diverse stimuli, including bacterial products and metabolic stressors, induce mtROS generation, which promotes NLRP3 oligomerization and subsequent caspase-1 activation (Feng et al., 2020). Crucially, mitochondrial DNA (mtDNA) released into the cytosol under conditions of mitochondrial damage can directly bind NLRP3 as a damage-associated molecular pattern, with non-oxidized mtDNA exhibiting higher binding affinity than oxidized forms (Fang et al., 2025). In NASH, palmitate-induced mtDNA release and subsequent mtDNA-NLRP3 complex formation constitute a key mechanistic link between lipotoxicity and inflammasome-driven inflammation (Ramos-Tovar and Muriel, 2023). This ROS generation therefore serves as a second messenger essential for NLRP3 inflammasome assembly (Harijith et al., 2014). Consequently, oxidative stress functions not merely as bystander damage but as a self-sustaining signaling mechanism that maintains inflammatory persistence through feedback loops involving antioxidant enzyme modulation (Harijith et al., 2014; Formanowicz, 2025).

**Figure 3:**
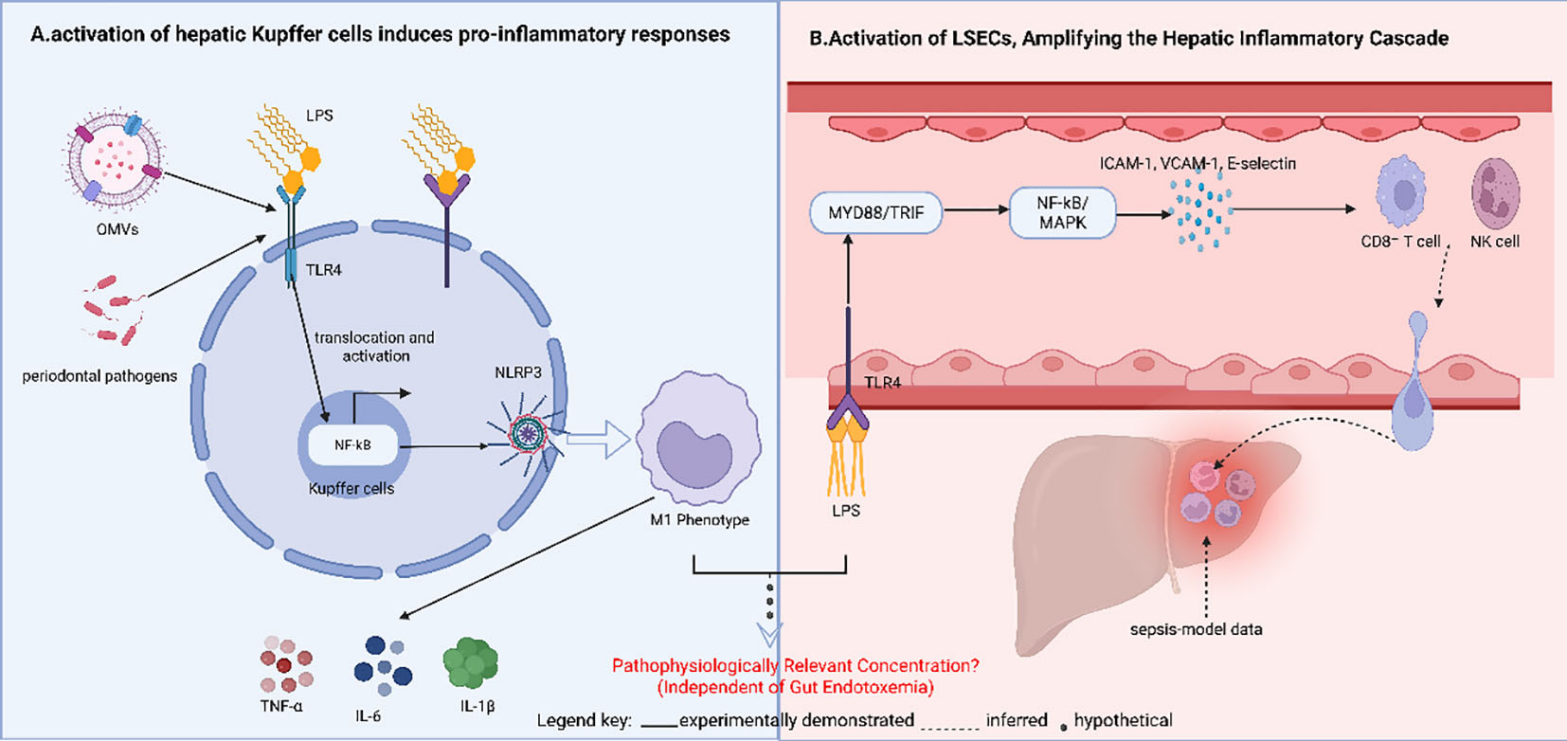
Direct activation of hepatic innate immunity and inflammatory cascades. Solid lines indicate experimentally demonstrated pathways; dashed lines indicate inferred pathways; dotted lines indicate hypothetical pathways. **(A)** activation of hepatic Kupffer cells induces pro-inflammatory responses. **(B)** Activation of LSECs, amplifying the hepatic inflammatory cascade. [Experimentally demonstrated in rodent models] Periodontal pathogens and their derivatives (LPS, OMVs) directly activate Kupffer cells via TLR4 and NLRP3 signaling pathways, inducing M1 macrophage polarization and the release of pro-inflammatory cytokines (TNF-α, IL-6, IL-1β) (Bellanti et al., 2025; Lv et al., 2025; Zhou et al., 2025). [Experimentally demonstrated *in vitro*] LPS stimulates LSECs via TLR4 signaling to upregulate adhesion molecules (ICAM-1, VCAM-1, E-selectin), promoting immune cell adhesion (Wang et al., 2025); [Inferred] the recruitment of peripheral immune cells (CD8+ T cells and NK cells) to the liver and subsequent amplification of the hepatic inflammatory response is inferred from sepsis-model data and awaits validation under chronic low-grade endotoxemia conditions. [Hypothetical] Whether these cascades operate at pathophysiologically relevant concentrations in human MASLD liver tissue, independent of gut-mediated endotoxemia, remains to be established. Legend key: — experimentally demonstrated; - - - inferred; ··· hypothetical.

Periodontal pathogen-derived products can also directly activate Kupffer cell gene expression: *Pg*-LPS suppresses MHC-II expression in Kupffer cells, concomitantly leading to impaired antigen presentation capacity (Wu et al., 2018); Gingipains inhibit the migration and phagocytic function of Kupffer cells/macrophages, thereby impairing inflammation resolution (Zhou et al., 2024); while Periodontal pathogen-derived OMVs reach the liver via the bloodstream, where they are internalized and activate Kupffer cells, stimulating the secretion of pro-inflammatory cytokines including IL-6, TNF-α, and MCP-1 to trigger an inflammatory cascade; simultaneously, they induce macrophage polarization toward the M1 pro-inflammatory phenotype, thereby potentially establishing a sustained inflammatory microenvironment (Lv et al., 2025). *In vivo* rodent studies demonstrated that following intraperitoneal injection of LPS-carrying *Pg* OMVs, the vesicles reached the liver via the bloodstream, where they accumulated and were predominantly internalized by Kupffer cells. Through activation of the TLR4/MyD88 signaling pathway, Kupffer cells released pro-inflammatory cytokines including IL-6, TNF-α, and IL-1β, exacerbating hepatic inflammation in these experimental models (Zhou et al., 2025). While these findings suggest that Kupffer cells may play a central mediating role in *Pg* OMV-induced liver injury under artificial exposure conditions, intraperitoneal injection does not replicate the chronic low-grade bacteremia of human periodontitis, and no human studies have demonstrated comparable OMV accumulation in hepatic tissue or quantified their contribution to MASH progression (**Figure 3A**).

Periodontal-local NLRP3 activation and systemic inflammatory propagation. Beyond hepatic effects, NLRP3 activation within periodontal tissues may contribute to systemic inflammatory burden. In patients with MASH and comorbid periodontitis, macrophages within gingival tissues exhibit elevated NLRP3 activation compared to lean controls (Inzaugarat et al., 2019). *In vitro*, palmitate and cholesterol—metabolic hallmarks of MASLD—synergistically enhance NLRP3 inflammasome activation in macrophages conditioned by *P. gingivalis* LPS or *E. coli* LPS, demonstrating that systemic metabolic dysfunction can “prime” periodontal immune cells for exaggerated inflammatory responses (Inzaugarat et al., 2019). Conversely, NLRP3-deficient mice infected with *P. gingivalis* exhibit altered gingival OPG mRNA expression and elevated peritoneal IL-6 levels, indicating that NLRP3 regulates both local periodontal and systemic immune responses to periodontal pathogens (Zhu et al., 2023). These findings suggest a bidirectional relationship: periodontal infection activates local NLRP3-driven inflammation, while metabolic comorbidities (obesity, dyslipidemia) prime the same pathway, potentially creating a self-amplifying cycle that propagates from oral cavity to liver via hematogenous dissemination.

Notably, no studies have examined whether *A. actinomycetemcomitans*, *T. forsythia*, or *T. denticola*—or polymicrobial consortia containing these species—activate Kupffer cells through the direct oral-hepatic pathway. *P. gingivalis* and *F. nucleatum* co-infection has been shown to accelerate colitis progression and induce systemic inflammation (Shirazi et al., 2020), but this study employed oral administration (permitting intestinal transit) and did not assess hepatic endpoints. The immunostimulatory profiles of polymicrobial periodontal communities versus individual species within the hepatic microenvironment therefore remain undefined.

#### 3.2.2 Direct activation of LSECs, amplifying the hepatic inflammatory cascade

LSECs constitute the first-line barrier of the hepatic vascular wall and are directly exposed to periodontal pathogens and their derivatives circulating in the bloodstream, serving as critical amplifiers of the hepatic inflammatory cascade (Wang and Liu, 2021; Mukherjee et al., 2025). *In vitro* studies demonstrate that periodontal pathogen *Fn* can directly activate LSECs via the TLR4-NF-κB signaling pathway, inducing the production of pro-inflammatory mediators (TNF-α and ROS) and leading to hepatic dysfunction in cell culture models (Mukherjee et al., 2025). Whether *Fn* or its products reach human LSECs at concentrations sufficient to trigger comparable responses *in vivo*, and whether this contributes to MASLD pathogenesis independent of gut-mediated endotoxemia, has not been established. *In vitro* studies have demonstrated that purified LPS, acting as the ligand of TLR4, can directly act on LSECs in culture. Through activation of MYD88-dependent and TRIF-dependent signaling pathways, and subsequent activation of NF-κB and MAPK signaling cascades, this stimulation significantly upregulates the expression of adhesion molecules on the cell surface, including CD54 (ICAM-1), CD106 (VCAM-1), and selectins. This, in turn, promotes the adhesion of peripheral blood leukocytes and lymphocytes (particularly CD8^+^ T cells and NK cells) to LSECs and their accumulation within the liver, recruiting peripheral immune cells to infiltrate the hepatic tissue, thereby amplifying local inflammatory responses in experimental systems (Wang et al., 2025). These mechanistic insights derive primarily from sepsis models employing supraphysiological LPS concentrations; their applicability to the chronic low-grade endotoxemia of periodontitis-associated MASLD remains uncertain. Moreover, all direct LSEC activation evidence pertains to *Pg*-LPS or *F. nucleatum* whole bacterial components (Mukherjee et al., 2025; Wang et al., 2025). Whether LPS from *A. actinomycetemcomitans*, *T. forsythia*, or *T. denticola*—or lipooligosaccharide variants with distinct lipid A structures—differentially modulate LSEC adhesion molecule expression and immune cell recruitment through the direct oral-hepatic pathway has not been investigated. The structural and immunological heterogeneity of periodontal pathogen LPS variants represents a critical knowledge gap limiting the generalizability of current mechanistic frameworks (**Figure 3B**).

#### 3.2.3 Emerging candidates: beyond Kupffer cells and LSECs

While dendritic cells (DCs) and innate lymphoid cells (ILCs) have been implicated in periodontal inflammation and general liver disease (Xu et al., 2022; Bourinet et al., 2023; Ma et al., 2023), direct evidence for their hepatic-specific activation by periodontal pathogens in MASLD remains elusive (Dioguardi et al., 2025). Future studies utilizing cell-specific depletion models are warranted to determine whether these populations represent bystanders or active participants in periodontitis-associated liver disease.

### 3.3 Proposed contribution to HSC activation and hepatic fibrosis progression: experimental hypotheses awaiting human validation

HSCs activation is the central event in hepatic extracellular matrix over-deposition and hepatic fibrogenesis (Lee and Friedman, 2011; Islam et al., 2025), while hepatic fibrosis represents the strongest independent predictor for the progression of MASLD patients toward cirrhosis, hepatocellular carcinoma, and all-cause mortality (Ekstedt et al., 2015). Current studies have shown that periodontal pathogens and their derivatives have been shown in experimental systems to induce HSCs activation through two distinct pathways namely direct effects and intrahepatic paracrine signaling both independent of intestinal mediation, thereby potentially contributing to MASLD-associated hepatic fibrosis progression (Nagasaki et al., 2020). It is critical to emphasize that all direct HSC activation evidence derives from *P. gingivalis* challenge models. The red complex (*Pg*, *T. forsythia*, *T. denticola*) and *F. nucleatum* exhibit synergistic inflammatory induction in periodontal ligament stem cells (Kendlbacher et al., 2024), but whether such polymicrobial synergy extends to hepatic stellate cell activation through hematogenous dissemination has not been examined. *A. actinomycetemcomitans*, *T. forsythia*, and *T. denticola* have no reported direct experimental evidence for HSC activation via the direct oral-hepatic pathway. The following mechanisms therefore represent Pg-specific proof-of-concept findings whose generalizability to polymicrobial periodontitis remains speculative.

It is critical to distinguish intrahepatic paracrine effects (secondary to direct bacterial stimulation of hepatocytes/Kupffer cells) from gut-mediated indirect effects (systemic endotoxemia via portal vein). Here, “paracrine” refers to local cytokine relay within the liver microenvironment, still constituting a putative direct pathway as defined in Chapter **2** (hematogenous dissemination without intestinal transit).

#### 3.3.1 Periodontal pathogens and their derivatives directly act on HSCs to promote their activation

*In vitro*, Pg has been shown to induce TGF-β1 autocrine production in immortalized or primary HSCs via gingipain-mediated activation of protease-activated receptor 2 (PAR2), while its LPS/lipoprotein has been shown to stimulate Galectin-3 (Gal-3) production in these cells. These findings establish proof-of-concept under controlled conditions; no human histopathological studies have demonstrated gingipain-mediated PAR2 activation or Gal-3 upregulation in HSCs within MASLD liver tissue. Gal-3 upregulates TGF-β receptor II expression to enhance sensitivity to TGF-β1. Together, these mechanisms synergistically may synergistically activate Smad and ERK signaling pathways in experimental systems, potentially promoting HSC differentiation into myofibroblasts with subsequent secretion of α-smooth muscle actin (α-SMA) and type I collagen (Nagasaki et al., 2020). While these findings establish that Pg products can directly activate HSCs under controlled conditions, no human histopathological studies have demonstrated gingipain-mediated PAR2 activation or Gal-3 upregulation in HSCs within MASLD liver tissue, and the contribution of this pathway to human fibrosis progression remains hypothetical. *In vivo* experiments employing a high-fat diet-induced MASH mouse model with odontogenic *P. gingivalis* infection demonstrated that the infection markedly induced hepatic crown-like structure (hCLS) formation and activated HSCs, characterized by elevated Gal-3 expression, increased phosphorylated Smad2 levels, and extracellular matrix deposition; local and/or systemic azithromycin administration eliminated the infectious foci, reduced hCLS counts, and suppressed HSC activation and hepatic fibrosis progression in these animals (Nagasaki et al., 2021). These findings establish proof-of-concept for a causal role of Pg in experimental fibrogenesis, but the odontogenic infection model employs bacterial loads and inflammatory stimuli that may exceed those of human chronic periodontitis, and no human interventional studies have assessed whether periodontal therapy reverses HSC activation or fibrosis stage in MASLD patients. However, P. gingivalis OMVs cannot directly activate HSCs, but instead promote their activation indirectly via paracrine mechanisms (Lv et al., 2026). Whether OMVs from *F. nucleatum*, *A. actinomycetemcomitans*, *T. forsythia*, or *T. denticola* exhibit direct HSC-activating capacity—or whether polymicrobial OMV mixtures produce synergistic fibrogenic effects—has not been investigated. The species-specificity of OMV-HSC interactions represents a significant gap given the polymicrobial nature of periodontal biofilms.

#### 3.3.2 Intrahepatic paracrine amplification of HSC activation

**Figure 4:**
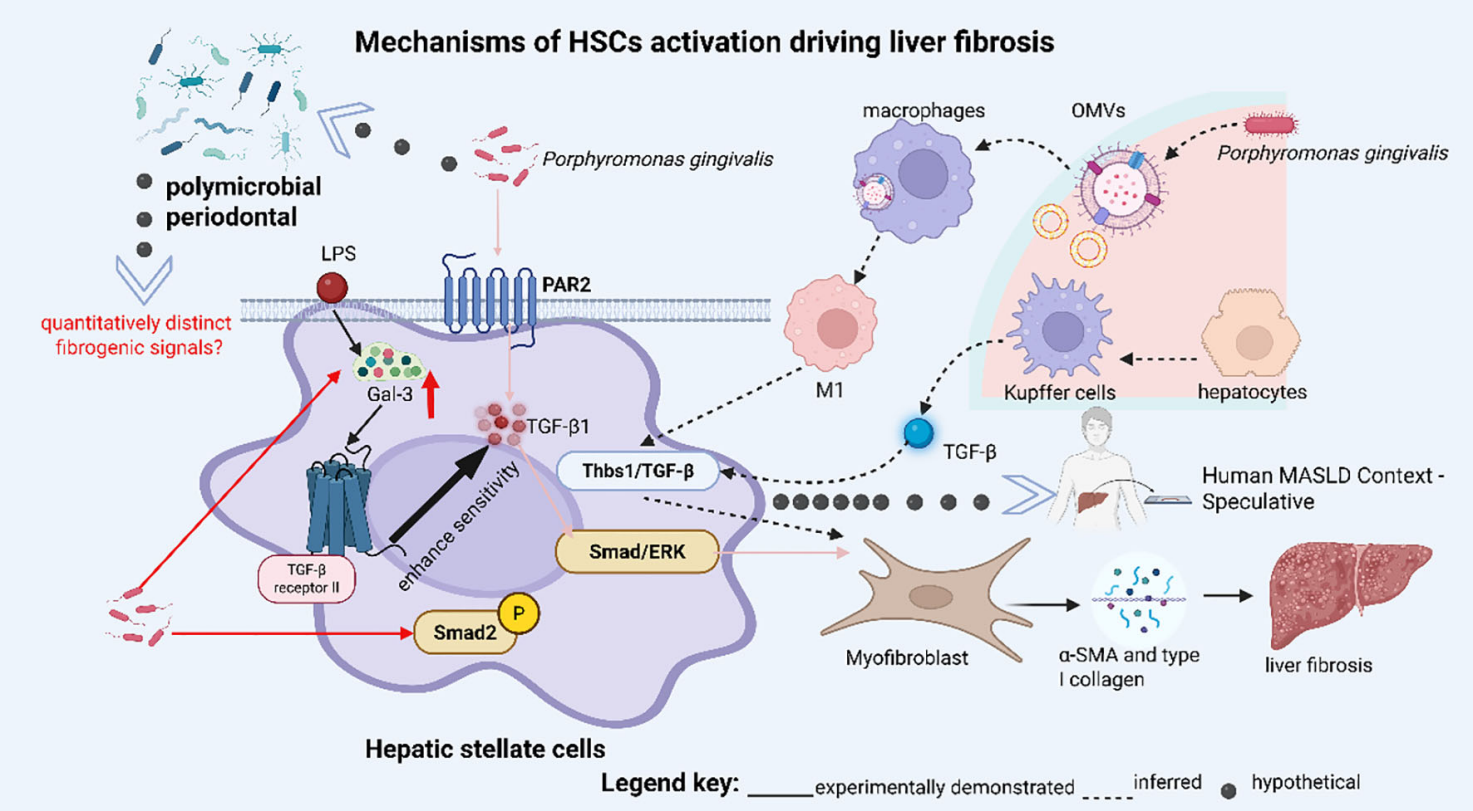
Mechanisms of HSCs activation driving liver fibrosis. Solid lines indicate experimentally demonstrated pathways; dashed lines indicate inferred pathways; dotted lines indicate hypothetical pathways. (1) [Experimentally demonstrated *in vitro*] *P. gingivalis* directly stimulates HSCs via gingipain-mediated PAR2 activation and LPS-induced Gal-3 upregulation, amplifying TGF-β1/Smad/ERK signaling to drive myofibroblast differentiation and extracellular matrix production (Nagasaki et al., 2020) (Nagasaki et al., 2021). (2) [Experimentally demonstrated in rodent models] *In vivo*, odontogenic P. gingivalis infection induces hepatic crown-like structure formation and activates HSCs, characterized by elevated Gal-3 expression and phosphorylated Smad2 (Nagasaki et al., 2021); azithromycin administration eliminates infectious foci and suppresses HSC activation in these models. (3) [Inferred] Bacterial OMVs and paracrine signals from hepatocytes and Kupffer cells may induce indirect HSC activation through macrophage M1 polarization and the Thbs1/TGF-β signaling axis (Seki et al., 2025; Lv et al., 2026); this represents intrahepatic paracrine amplification downstream of direct bacterial stimulation. (4) [Hypothetical] Whether comparable OMV-mediated paracrine activation occurs in human MASLD liver tissue, and whether polymicrobial periodontal challenge produces quantitatively distinct fibrogenic signals, remains entirely speculative. Legend key: — experimentally demonstrated; - - - inferred; ··· hypothetical.

As defined in the operational boundaries of this chapter, paracrine activation represents a downstream amplification mechanism of the direct pathway (Seki et al., 2025), distinct from gut-mediated indirect effects. In addition to direct effects, periodontal pathogens and their products may indirectly facilitate HSCs activation in the liver microenvironment through the regulation of multicellular paracrine networks (including, but not limited to, hepatocytes, Kupffer cells, and infiltrating macrophages) (Albuquerque-Souza and Sahingur, 2022; Lv et al., 2026). Existing studies have shown that *Pg*-stimulated hepatocytes secrete large amounts of pro-fibrotic cytokines and chemokines, and their supernatants significantly induce HSCs activation (Nagasaki et al., 2020); Kupffer cells activated by *Pg* derivatives have been shown to secrete key pro-fibrotic factors such as TGF-β, which may serve as potent paracrine stimuli for HSCs activation (Ying et al., 2026); NLRP3-driven IL-1β release from Kupffer cells represents an additional paracrine mechanism linking periodontal infection to HSC activation: IL-1β directly promotes HSC activation and collagen synthesis, while concurrently priming NLRP3 expression in surrounding cells through NF-κB signaling, thereby sustaining the inflammatory-fibrogenic microenvironment (Knorr et al., 2023). Upon uptake by macrophages, *P. gingivalis* OMVs induce M1 polarization and upregulate Thbs1 expression, thereby indirectly activating HSCs via the Thbs1/TGF-β signaling pathway and promoting hepatic fibrosis (Lv et al., 2026) (**Figure 4**). The paracrine amplification mechanisms described here are exclusively *Pg*-derived. Whether *F. nucleatum*, *A. actinomycetemcomitans*, *T. forsythia*, or *T. denticola* stimulate hepatocytes or Kupffer cells to secrete comparable profibrotic mediators, or whether polymicrobial challenge produces quantitatively or qualitatively distinct paracrine signals, remains entirely unknown. *P. gingivalis* and *F. nucleatum* co-infection models have demonstrated enhanced systemic inflammation (Nasiri et al., 2023), but hepatic fibrogenic endpoints were not assessed, and oral administration precluded distinction between direct and gut-mediated pathways.

### 3.4 Pathogenic vicious cycles: integrative network analysis

The foregoing sections have delineated distinct cellular targets of periodontal pathogens within the liver based predominantly on preclinical evidence. It must be re-emphasized that these mechanisms derive almost exclusively from monomicrobial *Pg* challenge models and may not reflect the pathogenic potential of polymicrobial periodontal communities. Periodontal biofilms exhibit complex ecological interactions—including metabolic cooperation between *Pg* and *T. forsythia*, bridging by *F. nucleatum*, and immunomodulation by *A. actinomycetemcomitans*—that may substantially alter virulence factor production, OMV cargo, and immunostimulatory profiles (Hajishengallis, 2015). While these processes have been characterized separately in experimental models, they may constitute self-amplifying vicious cycles potentially amplified by a redox-centric network. The following integrative framework synthesizes findings from *in vitro* and animal studies into interconnected positive feedback loops; these should be regarded as theoretically plausible mechanisms rather than established pathophysiology in human MASLD. This section synthesizes the direct mechanisms into interconnected positive feedback loops that operate alongside or in parallel with gut-mediated pathways.

#### 3.4.1 The steatosis-inflammation feed-forward loop

In preclinical models, the initial interaction between periodontal pathogen derivatives and hepatocytes may establish a lipotoxic-inflammatory axis that accelerates MASH-like pathology. As detailed in Section 3.1, *Pg*-LPS-induced mitochondrial dysfunction in experimental systems generates ROS (Wang et al., 2022; Ying et al., 2026), which may function as a metabolic modulator potentially stabilizing SREBP-1c via NF-κB/HDAC1 signaling (Guo et al., 2020; Prasun et al., 2021), thereby contributing to hepatic lipid accumulation in these models (Zhang et al., 2025) (Prasun et al., 2021). Beyond its direct metabolic effects, ROS—particularly mtROS—serves as the critical second signal for NLRP3 inflammasome assembly in Kupffer cells (Feng et al., 2020). In this framework, NLRP3 functions as a central convergence node integrating microbial stimulation (periodontal pathogen products), oxidative stress (mtROS/mtDNA), and innate immune activation (Inzaugarat et al., 2019). Whether this feed-forward loop operates in human MASLD, and whether periodontitis-derived ROS represents a quantitatively significant driver distinct from metabolic syndrome-associated oxidative stress, remains unknown. Concurrently, lipotoxic hepatocytes release damage-associated molecular patterns (DAMPs) and oxidized phospholipids, which activate Kupffer cells through TLR4 (Yue et al., 2021) and prime NLRP3 inflammasomes (Harijith et al., 2014; Zhang et al., 2019).

**Figure 5:**
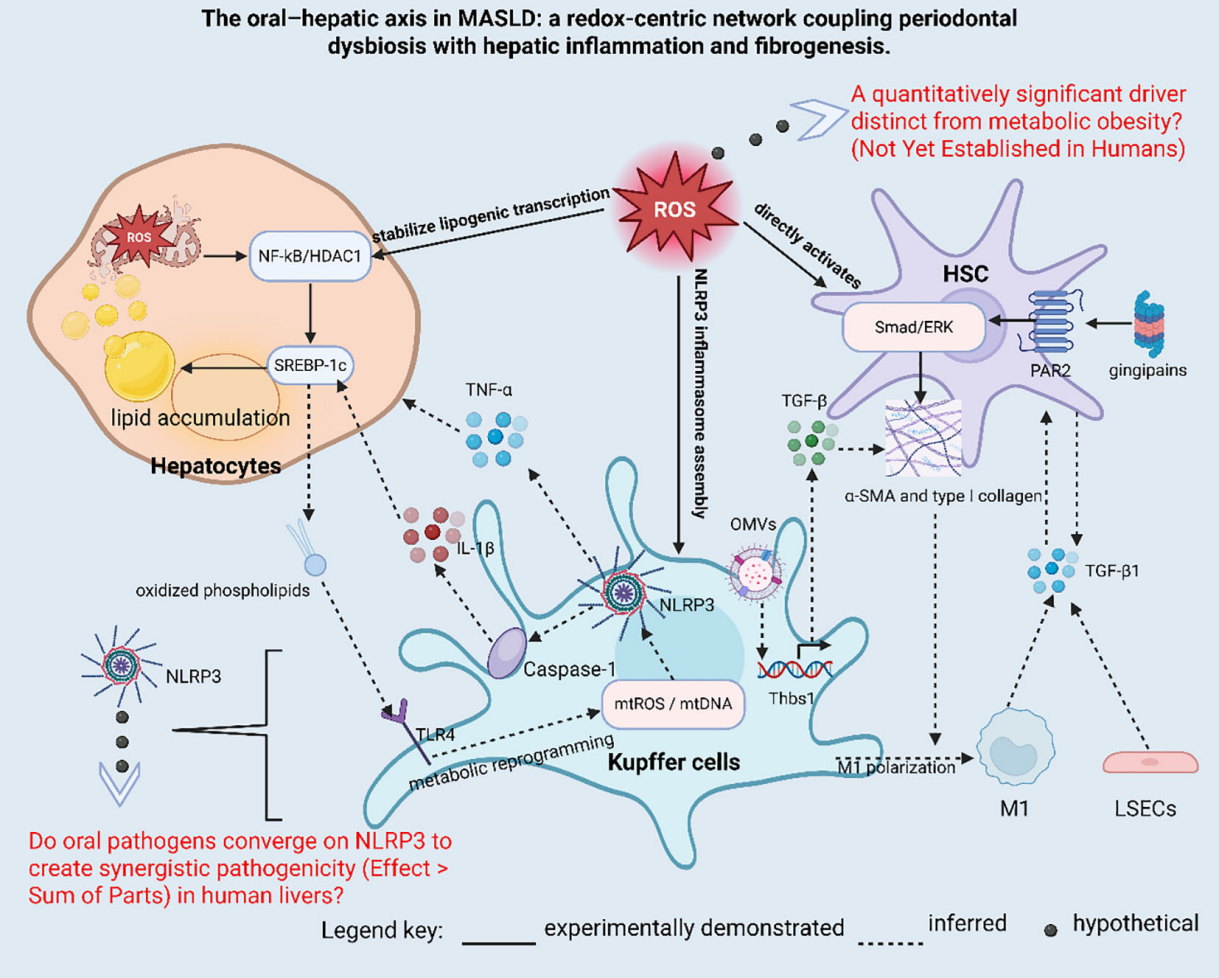
Redox-centric integration of pathogenic vicious cycles in MASLD. Solid lines indicate experimentally demonstrated pathways; dashed lines indicate inferred pathways; dotted lines indicate hypothetical pathways. This figure presents a hypothetical integrative framework synthesizing disparate preclinical observations into interconnected positive feedback loops. (1) [Experimentally demonstrated in preclinical models] ROS stabilizes lipogenic transcription (SREBP-1c via NF-κB/HDAC1) in hepatocytes (Guo et al., 2020; Prasun et al., 2021; Zhang et al., 2025), drives NLRP3 inflammasome assembly in Kupffer cells (Feng et al., 2020; Fang et al., 2025), and directly activates HSCs through redox-sensitive transcription factors (Bellanti et al., 2025). (2) [Inferred] NLRP3 serves as a mechanistic convergence node integrating microbial stimulation, metabolic stress (mtROS/mtDNA), and innate immune activation (Inzaugarat et al., 2019), potentially coupling steatosis and inflammation into a self-amplifying cycle. (3) [Hypothetical] The proposed redox-centric unification of steatosis-inflammation and inflammation-fibrogenesis loops into a single pathogenic network, wherein periodontal pathogen products converge on NLRP3 to create synergistic pathogenicity exceeding the sum of individual effects, represents a theoretical model awaiting systematic validation in human populations. (4) [Hypothetical] Whether periodontitis-derived ROS constitutes a quantitatively significant driver of hepatic redox dysregulation distinct from diet- or obesity-associated oxidative stress in human MASLD has not been established. Legend key: — experimentally demonstrated; - - - inferred; ··· hypothetical.

Once activated, Kupffer cells undergo metabolic reprogramming toward aerobic glycolysis (Zhang et al., 2024; Ma et al., 2025), generating substantial ROS (Bellanti et al., 2025; Lv et al., 2025) that provides the second signal for NLRP3 activation (Harijith et al., 2014). The NLRP3 inflammasome then drives caspase-1-mediated maturation of IL-1β and IL-18. IL-1β feeds back to hepatocytes to suppress PPARα-mediated fatty acid oxidation (Sasaki et al., 2018) while sustaining SREBP-1c activity (Guo et al., 2020; Prasun et al., 2021; Zhang et al., 2025); concurrently, IL-1β promotes further hepatocellular lipid accumulation and inflammatory cell recruitment (Knorr et al., 2023). IL-18 contributes to NK cell activation and IFN-γ production, amplifying the intrahepatic inflammatory milieu (Knorr et al., 2023). The resultant secretion of pro-inflammatory cytokines—particularly TNF-α and IL-1β (Lv et al., 2025; Zhou et al., 2025)—feeds back to hepatocytes (Nagasaki et al., 2020; Ying et al., 2026), suppressing PPARα-mediated fatty acid oxidation (Sasaki et al., 2018) while sustaining SREBP-1c activity (Guo et al., 2020; Prasun et al., 2021; Zhang et al., 2025). This may create a feed-forward loop wherein steatosis may drive inflammation, and inflammation, in turn, may exacerbate lipid accumulation through transcriptional reprogramming (Prasun et al., 2021; Zhang et al., 2025), with NLRP3 serving as the mechanistic hub that converts metabolic stress into sustained inflammatory signaling (Inzaugarat et al., 2019), potentially contributing to the pathological transition from simple steatosis to steatohepatitis (**Figure 5**).

#### 3.4.2 The inflammation-fibrogenesis lock-in mechanism

In experimental models, persistent inflammation within the hepatic microenvironment activates a paracrine signaling network (Seki et al., 2025) that may lock the liver into a profibrogenic state. M1-polarized Kupffer cells in rodent systems (Bellanti et al., 2025; Lv et al., 2025; Ma et al., 2025) and activated LSECs in culture (Wang and Liu, 2021; Zou et al., 2023; Mukherjee et al., 2025) secrete abundant TGF-β1 (Ying et al., 2026), which serves as the principal stimulus for HSC activation. The extent to which this paracrine amplification occurs in human MASLD liver tissue, and whether it is initiated or sustained by periodontitis-derived bacterial products as opposed to gut-mediated or metabolic stimuli, has not been determined. As detailed in Section **3.3.1**, *Pg*-derived gingipains further amplify this response by activating PAR2 on HSCs, promoting TGF-β1 autocrine signaling (Nagasaki et al., 2020), while Gal-3 upregulation enhances TGF-β receptor II sensitivity, maximizing Smad/ERK pathway activation (Nagasaki et al., 2020).

Critically, this pathway operates through intrahepatic paracrine amplification (Seki et al., 2025): OMVs internalized by macrophages induce Thbs1 expression (Lv et al., 2026), which activates latent TGF-β (Lv et al., 2026), creating a secondary wave of HSC activation that persists even after initial bacterial stimuli diminish. Once activated, HSCs reciprocally secrete extracellular matrix components (Nagasaki et al., 2020) and profibrotic mediators that maintain Kupffer cells in their M1 phenotype (Bellanti et al., 2025; Lv et al., 2025; Ma et al., 2025; Lv et al., 2026), preventing inflammatory resolution (**Figure 5**). This may establish a self-sustaining lock-in mechanism wherein inflammation may beget fibrosis, and fibrogenic signaling may perpetuate perpetuates the inflammatory milieu, potentially contributing to progression toward cirrhosis independent of continuous bacteremia (Seki et al., 2025; Lv et al., 2026).

#### 3.4.3 Redox-centric integration of pathogenic networks

Reactive oxygen species (ROS) have been proposed to function as a molecular integrator potentially connecting the steatosis-inflammation and inflammation-fibrogenesis cycles into a unified pathogenic network in preclinical models. Within this framework, the NLRP3 inflammasome emerges as a critical convergence node that transduces redox signals into inflammatory effector outputs (Inzaugarat et al., 2019). Specifically: (i) in hepatocytes, mitochondrial ROS stabilize lipogenic transcription factors (SREBP-1c via NF-κB/HDAC1) (Guo et al., 2020; Prasun et al., 2021; Zhang et al., 2025); (ii) in Kupffer cells, mtROS and cytosolic mtDNA serve as essential second signals for NLRP3 inflammasome assembly, driving IL-1β and IL-18 maturation (Feng et al., 2020; Fang et al., 2025); (iii) IL-1β, in turn, activates HSCs and promotes TGF-β1 expression, while TGF-β1 can feedback to prime NLRP3 expression in Kupffer cells via TAK1-NF-κB signaling, creating a self-sustaining inflammatory-fibrogenic loop (Knorr et al., 2023); (iv) oxidative stress directly activates HSCs through redox-sensitive transcription factors (Bellanti et al., 2025) and enhances TGF-β signaling (Nagasaki et al., 2020; Lv et al., 2026) (**Figure 5**). This redox-centric integration represents a theoretical framework synthesized from disparate experimental observations; it has not been validated as a unified pathogenic mechanism in human MASLD, nor has the specific contribution of periodontitis-associated ROS been isolated from other sources of hepatic oxidative stress in clinical populations.

This redox-centric integration may explain the temporal acceleration of MASLD in the context of periodontitis: while individual hits (bacterial LPS, OMVs, gingipains) initiate discrete cellular responses, NLRP3 serves as the mechanistic hub that couples these responses through ROS-mediated priming and activation, creating synergistic pathogenicity that exceeds the sum of individual effects (Harijith et al., 2014; Prasun et al., 2021; Bellanti et al., 2025; Zhang et al., 2025). In this model, periodontal pathogen products do not merely trigger parallel pathological processes; they converge on NLRP3 to activate an interconnected network of vicious cycles (Prasun et al., 2021; Seki et al., 2025; Zhang et al., 2025; Lv et al., 2026) that progressively may degrade hepatic homeostasis. The positioning of NLRP3 at the nexus of microbial stimulation, metabolic stress, and innate immune activation renders it a particularly attractive target for mechanistic investigation and potential therapeutic intervention (Inzaugarat et al., 2019).

However, it is critical to recognize that hepatocytes and Kupffer cells possess endogenous antioxidant defense systems capable of counteracting periodontitis-derived oxidative stress. The Nrf2/Keap1 pathway serves as the master regulator of cellular antioxidant responses: under basal conditions, Nrf2 is sequestered by Keap1 in the cytoplasm; upon oxidative challenge, Nrf2 translocates to the nucleus and binds antioxidant response elements (ARE) to upregulate heme oxygenase-1 (HO-1), NAD(P)H quinone oxidoreductase 1 (NQO1), and superoxide dismutase (SOD) (Wada et al., 2026). In periodontitis, gingival tissue Nrf2 expression is downregulated, accompanied by ROS accumulation and inflammatory cytokine elevation (Wada et al., 2026); conversely, pharmacological Nrf2 activation—exemplified by silibinin and epigallocatechin-3-gallate (EGCG)—suppresses NF-κB/NLRP3 signaling and attenuates oxidative damage and alveolar bone resorption (Fan et al., 2023; Li et al., 2023). Cross-organ proteomic analyses further reveal that periodontitis-driven hepatic oxidative stress is associated with dysregulated expression of lipid metabolism and antioxidant proteins, including HO-1, in the liver (Sun et al., 2025), suggesting that periodontal inflammation directly perturbs hepatic endogenous redox homeostasis. Notably, Porphyromonas gingivalis-derived OMVs, upon hematogenous dissemination to the liver, directly induce mitochondrial ROS burst and inhibit Nrf2 nuclear translocation in hepatocytes, thereby compromising hepatic antioxidant capacity and promoting lipid accumulation (Liu et al; Ying et al., 2026). Concurrently, mitochondrial ROS (mtROS) generated under periodontal hypoxic conditions serves as an essential upstream signal for NLRP3 inflammasome assembly in both gingival and hepatic tissues, establishing the mtROS-NLRP3 axis as a transduction hub linking local periodontal inflammation to systemic hepatic injury (Cheng et al., 2017; Isola et al., 2022; Vegda et al., 2024).

Beyond these mechanistic considerations, the ROS-centric integration model carries emerging translational relevance that warrants explicit discussion. While this framework remains theoretically constructed from disparate preclinical observations, therapeutic interventions targeting its core nodes have entered clinically relevant development, albeit with important limitations.

First, regarding Nrf2 activation as a pharmacological strategy, preclinical evidence supports the hepatoprotective potential of Nrf2 inducers in MASLD. Selective disruption of the NRF2-KEAP1 interaction has been shown to lead to NASH resolution and reduction of liver fibrosis in mouse models (Seedorf et al., 2022), and the Nrf2 activator bardoxolone methyl has been employed as a research tool in this context (Ciapała et al., 2023). However, clinical translation faces substantial hurdles: the phase 3 trial of bardoxolone methyl in diabetic chronic kidney disease was terminated prematurely due to cardiovascular safety signals, including increased heart failure and mortality (Gu et al., 2026), underscoring that systemic Nrf2 activation may carry unacceptable risks in comorbid populations. Natural Nrf2 activators-–including naringin, which alleviates periodontitis via direct AMPK/Nrf2 activation and concurrent NLRP3 inhibition (Chuanjian et al), and silibinin, which attenuates experimental periodontitis through downregulation of inflammation and oxidative stress (Li et al., 2023) offer potentially safer alternatives, though their bioavailability and dose-equivalence to human disease contexts remain unresolved (Tossetta et al., 2024; Wada et al., 2026).

Second, NLRP3 inflammasome inhibition represents a directly targetable node within the ROS-centric network. The selective NLRP3 inhibitor MCC950 has demonstrated efficacy in alleviating cholestatic liver injury and fibrosis in murine models (Adjei-Mosi et al., 2023; Wan et al., 2024), and dual intervention against oxidative stress and NLRP3 activation-–exemplified by thioacetamide-induced liver fibrosis models-–confirms the therapeutic rationale for simultaneous pathway targeting (Dwivedi et al., 2023). Nevertheless, MCC950’s clinical development was halted in phase II trials for rheumatoid arthritis due to hepatotoxicity concerns, suggesting that off-target effects at systemic doses may limit its therapeutic window (Wang et al., 2022). Emerging derivatives with improved safety profiles are under investigation, but no NLRP3 inhibitor has yet advanced to phase III for any liver indication.

Third, NOX-mediated ROS generation constitutes a druggable target upstream of both Nrf2 dysregulation and NLRP3 priming. The dual NOX1/4 inhibitor setanaxib (GKT137831) has progressed to phase 2b/3 clinical trials for primary biliary cholangitis (NCT05014672) and has demonstrated antifibrotic effects in preclinical liver models (Mansour et al., 2025). Its relevance to periodontitis-associated MASLD is further supported by evidence that Porphyromonas gingivalis disrupts endothelial cell NOX4/NRF2 balance (Wu et al., 2026), suggesting that NOX inhibition could theoretically intercept periodontitis-derived ROS at the source. However, setanaxib has not been evaluated in MASLD populations, and its effects on periodontal endpoints are unknown.

Finally, host-modulatory periodontal therapy offers a non-pharmacological approach that indirectly targets the ROS-NLRP3 axis. Non-surgical periodontal treatment reduces gingival crevicular fluid and serum levels of NLRP3, IL-1β, and caspase-1 (Shahbeik et al., 2021; Tayman and Kızılgün, 2025), while simultaneously attenuating systemic oxidative stress markers (Yang and Yang, 2025). The convergence of these effects on the same molecular nodes implicated in hepatic pathogenesis oxidative stress and inflammasome activation-–provides biological plausibility for periodontal intervention as a modulator of the ROS-centric network, even though direct hepatic biomarker changes post-therapy remains unproven.

In summary, while the ROS-centric integration model remains theoretically constructed, its constituent pathways are increasingly targetable by pharmacological and non-pharmacological interventions that have reached clinical development. However, none of these interventions have been evaluated specifically in patients with comorbid periodontitis and MASLD, and no trial has employed the direct oral-hepatic pathway as an explicit mechanistic framework. The translational relevance of this model therefore lies not in current clinical applicability, but in its capacity to guide future comorbidity-specific trial design particularly combination approaches that simultaneously modulate periodontal disease activity and hepatic redox-inflammatory status.

Beyond endogenous defenses, emerging therapeutic strategies targeting the redox-NLRP3 axis offer potential avenues for interrupting the periodontitis-MASLD pathogenic network. Natural compounds such as naringin activate AMPK/Nrf2 signaling while concurrently inhibiting NLRP3, attenuating alveolar bone resorption in periodontitis models; its metabolites may exert systemic hepatoprotective effects via circulation (Chuanjian et al). Dioscin inhibits NLRP3 activation by regulating K^+^ homeostasis and mitochondrial function, mitigating periodontal bone loss and suggesting potential hepatic applicability given the shared NLRP3 dependence of liver inflammation (Jiang et al., 2024). At the pharmacological level, 1-O-acetylbritannilactone (ABL) covalently modifies NLRP3 at Cys669 to disrupt the NLRP3-NEK7 complex formation, ameliorating high-fat diet-induced hepatic steatosis and fibrosis in preclinical models (Jin et al., 2026); complementary localized interventions, including Er: YAG laser biostimulation and pH-responsive hydrogel delivery systems, have demonstrated anti-inflammatory and tissue-protective efficacy in periodontal models (Ng et al., 2024; Xia et al., 2025), suggesting that multimodal NLRP3-modulatory strategies may be applicable across oral and hepatic contexts. Host-modulatory periodontal therapy, specifically non-surgical periodontal treatment, significantly reduces gingival crevicular fluid and serum levels of NLRP3, IL-1β, and caspase-1, confirming that local intervention can systemically attenuate oxidative-inflammatory burden (Shahbeik et al., 2021; Tayman and Kızılgün, 2025). Nevertheless, these strategies have been evaluated predominantly in isolated disease models; rigorous clinical trials assessing combined Nrf2 activation and NLRP3 inhibition in patients with comorbid periodontitis and MASLD are currently unavailable.

## 4 Emerging clinical evidence of periodontal intervention in MASLD

The mechanisms outlined in Chapter 2 posit that periodontal pathogens and their derivatives reach the liver via systemic circulation, directly affecting hepatocyte lipid metabolism, activating innate immune responses, and promoting HSCs activation. This chapter examines whether periodontal intervention can disrupt these pathogenic processes and improve MASLD-related clinical outcomes. It must be stated explicitly that the clinical evidence presented herein is predominantly hypothesis-supporting rather than confirmatory, and rests almost exclusively on a single small-scale randomized controlled trial (RCT) by Kamata et al. (2022) (2022; n = 40) that directly examined periodontal intervention in patients with biopsy-proven MASLD. All other available clinical studies were conducted in non-MASLD populations (e.g., chronic periodontitis, localized aggressive periodontitis, or chronic kidney disease with periodontitis) and therefore provide only indirect, mechanistically supportive evidence. No clinical studies have directly demonstrated reversal of hepatic fibrosis following periodontal therapy, and mechanistic biomarkers linking periodontal treatment with hepatic improvement remain largely unavailable. Consequently, the clinical evidence base for interrupting the direct oral--hepatic pathway in MASLD remains preliminary and should be interpreted as generating hypotheses for future investigation rather than establishing clinical efficacy. **Table 3** summarizes both this single direct RCT and the indirect studies, detailing their designs, sample characteristics, intervention protocols, follow-up durations, and key findings across hepatic metabolic, inflammatory, and fibrotic endpoints.

**Table 3:** Clinical evidence and methodological quality of periodontal intervention: direct evidence from MASLD patients and indirect mechanistic support from non-MASLD populations.

Study	Design	Sample size	Population characteristics	Intervention	Follow-up duration	Primary endpoints	Key findings	Mechanism linkage	Evidence quality	Reference
Kamata et al., 2022	Multicenter randomized controlled trial	40	Biopsy-proven MASLD with moderate-to-severe periodontitis	SRP	12–60 weeks	Decreased CAP; decreased MRI-PDFF; decreased ALT/AST	Significant reduction in hepatic fat content (CAP↓), improved liver inflammation, sustained effect at 60 weeks	Directly validates lipid metabolism mechanisms and inflammatory mechanisms	High (low risk of bias RCT)	(Kamata et al., 2022)
Ide et al., 2004	Prospective interventional cohort	15	Chronic periodontitis patients	SRP	Baseline, 15 min, 1 h, 2 h	Plasma endotoxin; serum TNF-α, IL-6, hs-CRP	Immediate post-SRP endotoxin borderline increase (*P* = 0.061), significant TNF-α (*P* = 0.0387) and IL-6 (*P* < 0.0001) elevation	Validates acute Kupffer cell activation and cytokine release	Moderate (no control group, small sample)	(Ide et al., 2004)
Kalash et al., 2015	Randomized controlled trial	12	Localized aggressive periodontitis	SRP + amoxicillin 500 mg + metronidazole 250 mg (TID × 7 days)	3 months, 12 months	Serum endotoxin; clinical periodontal parameters	Significant endotoxin reduction at 3 months, sustained suppression at 12 months, positively correlated with clinical improvement	Validates chronic endotoxin-mediated metabolic and inflammatory mechanisms	Moderate (small sample, open-label)	(Kalash et al., 2015)
Lafaurie et al., 2011	Prospective observational	30	Severe generalized periodontitis	SRP (full-mouth)	Baseline, immediate	Blood *Pg*-DNA detection rate (16S rRNA)	Baseline 16.6% patients *Pg*-DNA positive, increased to 54.8% immediately post-SRP	Validates mechanical bacteremia and hematogenous dissemination route	Moderate (observational, no control)	(Lafaurie et al., 2011)
Zhang et al., 2021	Randomized controlled trial	30	Chronic periodontitis	Full-mouth SRP	Baseline, 1 week, 6 weeks	Blood microbiome (16S rRNA sequencing)	Microbiome diversity decreased at 1 week post-SRP, recovered to baseline at 6 weeks	Supports physiological reversibility and clinical significance of hematogenous route	Moderate (no sham surgery control)	(Zhang et al., 2021)
Chaudhry et al., 2022	Single-arm before-after control	45	Chronic kidney disease with chronic periodontitis	Non-surgical periodontal therapy	3 months	Periodontal parameters; hs-CRP; IL-6; renal function indicators	Significant reduction in hs-CRP and IL-6 following periodontal treatment	Supports systemic inflammation improvement mechanisms	Low (single-arm design, multiple confounders)	(Chaudhry et al., 2022)

Evidence quality assessment based on Cochrane Risk of Bias tool: High, low risk of bias randomized controlled trial; Moderate, RCT with design limitations or high-quality observational studies; Low, single-arm trials, case series, or expert opinion. Direct MASLD evidence: only Kamata et al. (2022) enrolled biopsy-proven MASLD patients. All other studies represent indirect evidence from non-MASLD populations; they provide mechanistic plausibility for the direct pathway but do not establish direct clinical efficacy in MASLD.

### 4.1 Effects on hepatic lipid metabolism and steatosis

The evidence supporting improvement of hepatic steatosis after periodontal therapy relies predominantly on a single randomized clinical trial. To date, only one small-scale RCT (Kamata et al., n = 40) has directly examined whether non-surgical periodontal therapy (SRP) reduces hepatic steatosis in patients with comorbid periodontitis and biopsy-proven MASLD (Kamata et al., 2022). This multicenter study reported that SRP significantly decreased controlled attenuation parameter (CAP) and magnetic resonance imaging-proton density fat fraction (MRI-PDFF) at 12 weeks compared to tooth brushing alone, with sustained reductions in liver fat content maintained at 60 weeks. These imaging parameters directly reflect hepatic triglyceride content (Kamata et al., 2022), corresponding to the lipid accumulation mechanisms described in Section **3.1**. The reduction in hepatic steatosis following periodontal therapy is associated with decreased circulating levels of periodontal virulence factors (Kamata et al., 2022). However, these findings from a single, small trial should be regarded as hypothesis-generating; independent replication in larger, adequately powered RCTs is required before any definitive conclusions regarding clinical efficacy can be drawn.

Indirect evidence from non-MASLD populations provides insight into the temporal dynamics of systemic bacterial translocation following SRP. Although these studies did not enroll MASLD patients, they inform the mechanistic framework by which periodontal intervention might modulate the direct oral–hepatic pathway. Mechanical instrumentation during SRP induces transient bacteremia and endotoxemia through ulcerated periodontal pocket epithelium, with *Pg* DNA detectable in peripheral blood within 24 hours post-treatment as demonstrated by 16S rRNA gene sequencing analysis (Zhang et al., 2021). Specifically, Ide et al. (2004) reported that plasma endotoxin levels exhibited borderline significant increases (*P* = 0.061) accompanied by significant elevations in TNF-α (*P* = 0.0387) and IL-6 (*P* < 0.0001) during the immediate post-SRP period (15–120 minutes), indicating acute systemic inflammatory responses to bacterial and LPS translocation (Ide et al., 2004).

In non-MASLD cohorts, longitudinal investigations reveal divergent outcomes based on periodontal disease phenotype and therapeutic protocol. In patients with localized aggressive periodontitis receiving comprehensive therapy combining SRP with systemic antibiotics (amoxicillin 500 mg plus metronidazole 250 mg t.i.d. for 7 days), serum endotoxin levels demonstrated significant reductions at 3-month follow-up, with sustained suppression observed at 12 months that correlated positively with clinical improvements in probing depth and clinical attachment loss (Kalash et al., 2015). Conversely, untreated periodontal disease maintains chronic low-grade endotoxemia, with baseline detection of P. gingivalis DNA in peripheral blood of 16.6% of patients with severe generalized periodontitis, increasing to 54.8% immediately following SRP procedures (Lafaurie et al., 2011).

Similarly, in non-MASLD patients, microbial community stability in systemic circulation recovers within 6 weeks following full-mouth SRP, with blood microbial diversity returning to pretreatment levels despite initial perturbation (Zhang et al., 2021). These findings collectively indicate that while SRP transiently elevates circulating P. gingivalis DNA and LPS through mechanical dissemination of periodontal pathogens, complete periodontal therapy—particularly when augmented with systemic antimicrobial agents—ultimately reduces systemic endotoxin burden and inflammatory markers (hs-CRP, IL-6) over extended follow-up periods (Chaudhry et al., 2022). Given that *Pg-*LPS directly upregulates acetyl-CoA carboxylase 1 (ACC1) and enhances fatty acid metabolism, contributing to hepatic lipid accumulation (Sasaki et al., 2018; Ding et al., 2019), the reduction in circulating LPS observed in non-MASLD cohorts may plausibly contribute to improved hepatic lipid homeostasis in MASLD, but this remains speculative. Moreover, no clinical studies in MASLD patients have simultaneously measured changes in circulating *Pg-*LPS and hepatic lipid content to establish a direct dose-response relationship.

### 4.2 Effects on hepatic inflammation

Within the limited direct evidence, the same MASLD-specific RCT (Kamata et al., n = 40) also reported significant decreases in serum ALT and AST levels following SRP (Kamata et al., 2022). These improvements in liver enzymes suggest attenuation of hepatocellular injury (Kamata et al., 2022), potentially corresponding to the reduction in Kupffer cell activation and inflammatory cytokine release described in Section **3.2**.

The inflammatory improvements in this single MASLD RCT may be partially mediated by reduced endotoxemia. This multicenter RCT reported that SRP decreased serum endotoxin levels at 12 weeks in MASLD patients, with concomitant improvements in liver function tests (Kamata et al., 2022). Since endotoxins activate Kupffer cells via TLR4/NF-κB signaling, this reduction aligns with the proposed mechanism. However, this study did not measure hepatic cytokine levels or Kupffer cell activation markers, leaving the mechanistic link inferential rather than demonstrated. No other MASLD-specific clinical trial has independently validated these findings.

### 4.3 Effects on hepatic fibrosis

No clinical studies have directly demonstrated reversal of hepatic fibrosis following periodontal therapy. Evidence regarding periodontal intervention effects on hepatic fibrosis in MASLD patients remains extremely limited. The same single small RCT (Kamata et al., n = 40) utilizing liver stiffness measurement (LSM) by transient elastography reported significant improvements in LSM values following non-surgical periodontal therapy (Kamata et al., 2022). This finding suggests potential attenuation of fibrogenesis, which would correspond to the HSC activation mechanisms described in Section **3.3**.

However, LSM is influenced by both inflammation and fibrosis, and no clinical trials in MASLD or non-MASLD populations have employed liver biopsy to assess changes in fibrosis stage or HSC activation markers (e.g., α-SMA, TGF-β1) following periodontal intervention. Consequently, whether periodontal therapy directly reverses hepatic fibrosis—or merely reduces inflammation-associated stiffness—remains entirely unresolved in the clinical literature. The direct effect of periodontal therapy on HSC activation, as described in Section **3.3.1**, therefore remains unverified in human studies and has not been specifically tested in MASLD patients.

In summary, the clinical evidence directly linking periodontal intervention to improved MASLD outcomes via the direct oral--hepatic pathway remains preliminary and rests almost exclusively on a single small RCT (n = 40). This limited evidence base should be interpreted as hypothesis-supporting rather than confirmatory: (i) improvement of hepatic steatosis after periodontal therapy has been reported in only one RCT, requiring independent replication; (ii) no clinical study has directly demonstrated reversal of hepatic fibrosis; and (iii) mechanistic biomarkers linking periodontal treatment with hepatic improvement remain largely unavailable. The remaining cited studies, while providing valuable mechanistic insights into endotoxemia reduction, systemic inflammation, and bacteremia dynamics, were conducted in non-MASLD populations and therefore cannot establish direct efficacy in MASLD. This profound evidence gap underscores the urgent need for large-scale, MASLD-specific randomized controlled trials—with mechanistic substudies incorporating biomarker assessment and liver biopsy for fibrosis staging—to validate whether interrupting periodontal infection can meaningfully alter the course of direct oral--hepatic MASLD pathogenesis.

## 5 Critical appraisal and standardized research framework

Despite accumulating evidence for direct periodontal–hepatic pathways, the field faces three fundamental constraints that impede clinical translation.

First, the causal inference gap. Current evidence relies predominantly on monomicrobial Pg models, which fail to recapitulate the polymicrobial nature of periodontitis. Although *F. nucleatum* has been implicated in TLR4-mediated hepatic inflammation (Mukherjee et al., 2025) and serum antibody responses to A. actinomycetemcomitans correlate with metabolic abnormalities in MASLD patients (Hatasa et al., 2021), no experimental studies have directly compared the hepatic pathogenicity of *Pg*, *Fn*, *Aa*, *Tf*, and *Td*—or their polymicrobial combinations—using gut-interference controls. Polymicrobial infection models that have demonstrated intravascular dissemination of periodontal pathogens to extrahepatic sites (Farrugia et al., 2022) have not assessed liver-specific endpoints. The ecological interactions within periodontal biofilms (e.g., co-aggregation, quorum sensing, metabolic cross-feeding) may profoundly influence the quantity, quality, and immunostimulatory potential of bacteria and their derivatives entering the bloodstream (Hajishengallis, 2015), yet their impact on direct oral-hepatic pathogenesis remains entirely unexplored. Tail-vein and jugular-vein injection studies introduce bacteria directly into the systemic venous circulation, thereby routing them necessarily through the hepatic artery and bypassing both the oral barrier’s selective pressure and the portal venous system. While these models establish proof-of-concept for hematogenous dissemination, they cannot quantify the relative contribution of the hepatic artery versus portal venous recirculation under physiological conditions. Gingival inoculation models more closely mimic natural bacteraemia, yet without portal-vein versus systemic blood sampling, enteric diversion, or germ-free gut-interference controls, they cannot definitively distinguish whether bacteria detected in the liver arrived via the hepatic artery, via portal venous recirculation following transient intestinal transit, or through both routes simultaneously. Moreover, existing Mendelian randomization studies assess periodontitis–MASLD association without stratifying for direct versus gut-mediated pathways, rendering them inadequate for causal deconvolution. Future studies must adopt strain-resolved metagenomics paired with portal-vein catheterization in gut-interference models to isolate and quantify the relative contribution of each route. Future studies must adopt strain-resolved metagenomics to quantify oral-derived bacteria in liver tissue, complemented by portal-vein catheterization models in germ-free animals to isolate direct hepatic effects from intestinal confounders.

Second, mechanistic fragmentation. The field has prioritized single-cell-type, single-pathway analyses (e.g., *Pg*-LPS→TLR4→NF-κB in hepatocytes) while neglecting intercellular networks. Notably, the relative contribution of direct bacterial effects versus intrahepatic paracrine amplification remains unquantified. We propose a multi-omics integration framework: spatial transcriptomics to map bacterial distribution within liver lobules, combined with single-cell RNA-seq to dissect Kupffer cell–hepatocyte–HSC crosstalk. Human liver organoids co-cultured with periodontal OMVs should validate rodent findings.

Third, clinical evidence limitations. To date, only one small-scale RCT (n = 40) has directly examined periodontal intervention in MASLD patients; all other clinical evidence is indirect, derived from non-MASLD populations. Existing direct-evidence RCTs rely on surrogate endpoints (CAP, ALT) that lack prognostic specificity for hard outcomes (fibrosis progression, HCC). The transient bacteremia post-scaling introduces noise that may obscure long-term benefit. Consequently, the efficacy of periodontal intervention specifically in MASLD remains unvalidated, and the generalizability of current findings to broader MASLD populations is highly uncertain. Standardized protocols must specify: (i) adjunctive systemic antibiotics for aggressive periodontitis phenotypes; (ii) 12-month minimal follow-up to capture hepatic remodeling; (iii) liver biopsy substudies for fibrosis staging; and (iv) explicit enrollment of MASLD cohorts with biopsy-confirmed disease staging to generate direct, generalizable evidence. Additionally, targeted interventions (e.g., *Pg*-specific phage therapy) should enter preclinical development to test the direct-pathway hypothesis mechanistically.

Fourth, therapeutic evidence gaps. While preclinical studies have identified promising Nrf2 activators and NLRP3 inhibitors capable of attenuating periodontal inflammation and hepatic steatosis in isolated models (Chuanjian et al; Jiang et al., 2024; Jin et al., 2026), no randomized controlled trials have evaluated these agents in patients with comorbid periodontitis and MASLD. The translational potential of host-modulatory periodontal therapies—including non-surgical periodontal treatment that reduces local and systemic NLRP3/IL-1β burden (Shahbeik et al., 2021; Tayman and Kızılgün, 2025)—for direct hepatic benefit remains entirely speculative. Furthermore, the efficacy of natural antioxidants (e.g., naringin, EGCG) in simultaneously ameliorating oral and hepatic endpoints via the direct oral-hepatic pathway has not been prospectively tested (Chuanjian et al; Fan et al., 2023). Future clinical trial designs should incorporate mechanistic substudies to determine whether pharmacological or periodontal host-modulatory interventions can simultaneously modulate gingival crevicular fluid and hepatic biomarkers of redox stress and inflammasome activation.

Methodological standardization is urgent. Current animal models employ heterogeneous bacterial strains (ATCC 33277 vs. W83), inoculum doses (10^6^–10^8^ CFU), and detection modalities (qPCR vs. 16S rRNA), precluding meta-analysis. We advocate: (i) reference strains with whole-genome sequencing; (ii) mandatory gut-interference controls (germ-free or portal-vein injection); (iii) minimum reporting standards (bacterial viability assays, endotoxin quantification). Adoption of these standards by consortia (e.g., EASL-AAP collaboration) would enable cross-study validation essential for field maturation.

### 5.1 Reverse causality, shared risk factors, and residual confounding

An important limitation that warrants explicit acknowledgment is that periodontitis and MASLD share multiple established risk factors, including obesity, type 2 diabetes mellitus, insulin resistance, smoking, dietary habits, and socioeconomic determinants (Åberg and Helenius-Hietala, 2022). These shared risk factors generate substantial potential for residual confounding in observational studies: the observed associations may reflect common systemic inflammatory and metabolic backgrounds rather than direct pathogen-mediated effects alone (Åberg and Helenius-Hietala, 2022; Juzbašić et al., 2025).

Furthermore, the possibility of reverse causality cannot be excluded. MASLD-associated systemic inflammation and immune dysregulation may compromise oral barrier integrity and exacerbate periodontal inflammation, creating a bidirectional relationship (Åberg and Helenius-Hietala, 2022). Mendelian randomization analyses provide some protection against confounding by shared genetic and early-life environmental factors (Tan et al., 2024); however, existing studies have not stratified for direct versus gut-mediated pathways, rendering them inadequate for causal deconvolution of the specific oral-hepatic axis under review.

Consequently, the associations reported in observational and preclinical studies should be interpreted as consistent with-–but not demonstrative of-–a causal role for direct oral-hepatic bacterial transmission in MASLD pathogenesis.

## 6 Conclusion

This review synthesizes evidence predominantly from preclinical models (*in vitro* studies, rodent experiments, and bacterial tracer investigations) consistent with the hypothesis that periodontal pathogens may reach the liver via systemic circulation, potentially complementing or bypassing gut-mediated routes. we delineate a putative anatomical route from periodontal pocket to hepatic sinusoid and propose three mechanisms that may contribute to MASLD progression in experimental systems: potential modulation of hepatocyte lipid metabolism via TLR4/NF-κB signaling, potential activation of Kupffer cells and LSECs potentially promoting innate immune-inflammatory cascades, and potential induction of HSC activation through direct and intrahepatic paracrine pathways. It is critical to emphasize that these proposed mechanisms remain biologically plausible but clinically unvalidated hypotheses; they have been demonstrated under controlled experimental conditions but have not been confirmed to operate at pathophysiologically relevant levels in human MASLD liver tissue. It is important to note that this mechanistic framework almost exclusively reflects the role of *Porphyromonas gingivalis*, given its disproportionate representation in the current preclinical literature. *F. nucleatum* has been shown to activate TLR4-NF-κB/MAPK signaling in hepatic cells (Mukherjee et al., 2025) and promote oxidative stress and Kupffer cell activation (Cao et al., 2022), but these studies lack gut-interference controls and do not establish direct hematogenous dissemination to the liver. Serum antibody titers against *A. actinomycetemcomitans* correlate with metabolic abnormalities in MASLD patients (Hatasa et al., 2021), but this observational evidence cannot distinguish direct from gut-mediated effects or establish causality. *T. forsythia* and *T. denticola* have no reported direct experimental evidence for hepatic pathogenicity via the oral-hepatic pathway. Polymicrobial infection models incorporating these species have demonstrated intravascular dissemination (Zwickl et al., 2020), but hepatic endpoints were not assessed. The extrapolation of *Pg*-centric findings to the polymicrobial nature of human periodontitis therefore remains to be validated, and represents one of the most critical gaps limiting the clinical translation of the direct oral-hepatic pathway hypothesis. Furthermore, the vast majority of mechanistic studies employ single-species, single-virulence-factor experimental designs that do not recapitulate the complex microbial community dynamics and host immune responses characteristic of human periodontal disease. These pathways should be regarded as biologically plausible but clinically unvalidated hypotheses.

Preclinical studies suggest that these processes may form self-amplifying vicious cycles that operate alongside, or in parallel with, gut-mediated pathways. ROS may function as an integrative node connecting steatosis–inflammation and inflammation–fibrogenesis loops, potentially contributing to accelerated MASLD progression in experimental models in the context of periodontitis. Clinical evidence remains predominantly hypothesis-supporting rather than confirmatory. Preliminary evidence from a single small randomized controlled trial (n = 40) suggests that periodontal intervention may improve hepatic steatosis and inflammatory markers in patients with comorbid periodontitis and MASLD (Kamata et al., 2022); however, this study did not distinguish direct oral-hepatic effects from gut-mediated pathways, did not measure hepatic bacterial colonization or virulence factor levels, and employed surrogate endpoints (CAP, ALT) rather than histopathological or hard clinical outcomes. No clinical study has directly demonstrated reversal of hepatic fibrosis, and mechanistic biomarkers linking periodontal treatment with hepatic improvement remain largely unavailable. These findings therefore require independent replication in larger, adequately powered randomized controlled trials—with mechanistic substudies incorporating biomarker assessment and liver biopsy for fibrosis staging—before any translational relevance can be assessed.

However, causal inference remains severely limited by: (i) monomicrobial experimental models that fail to recapitulate the polymicrobial complexity of human periodontitis; (ii) artificial delivery routes (intravenous injection, intraperitoneal administration) that bypass the oral barrier and do not replicate chronic low-grade bacteremia; (iii) lack of gut-interference controls in gingival inoculation studies, preventing definitive discrimination between hepatic artery and portal venous delivery; and (iv) absence of human interventional studies capable of isolating direct oral-hepatic effects from gut-mediated pathways. Future research must adopt strain-resolved metagenomics of human liver tissue, multi-omics integration across experimental and clinical samples, and rigorous gut-interference controls (e.g., germ-free animals, portal-vein catheterization) to quantify the relative contribution of direct versus indirect pathways. Until such evidence is obtained, the proposed direct oral-hepatic pathway should be regarded as a compelling but unvalidated hypothesis. Addressing these gaps will advance oral–systemic health management and inform targeted MASLD prevention strategies.

Moreover, the therapeutic landscape for interrupting the direct oral-hepatic pathway remains in its infancy. Endogenous antioxidant systems, particularly the Nrf2/Keap1/ARE axis, represent critical host-modulatory mechanisms that may be pharmacologically reinforced to counteract periodontitis-derived hepatic oxidative stress (Li et al., 2023; Wada et al., 2026). Concurrently, NLRP3-targeted interventions—including natural compounds (naringin, dioscin) and small-molecule inhibitors (ABL)—have demonstrated preclinical efficacy in attenuating both periodontal bone loss and hepatic steatosis (Chuanjian et al; Jin et al., 2026), yet their clinical translation awaits validation in comorbidity-specific trials. Host-modulatory periodontal therapy, through reduction of local and systemic NLRP3/IL-1β burden, offers a non-pharmacological entry point (Shahbeik et al., 2021; Tayman and Kızılgün, 2025), though its direct impact on MASLD histopathology remains unproven. Addressing these therapeutic evidence gaps will be essential for advancing from mechanistic hypothesis to clinical intervention in periodontitis-associated MASLD.
